# Wheat Drought-Responsive Grain Proteome Analysis by Linear and Nonlinear 2-DE and MALDI-TOF Mass Spectrometry

**DOI:** 10.3390/ijms131216065

**Published:** 2012-11-29

**Authors:** Shan-Shan Jiang, Xiao-Na Liang, Xin Li, Shun-Li Wang, Dong-Wen Lv, Chao-Ying Ma, Xiao-Hui Li, Wu-Jun Ma, Yue-Ming Yan

**Affiliations:** 1 College of Life Science, Capital Normal University, Beijing 100048, China; E-Mails: jianghuang20051209@163.com (S.-S.J.); liangxiaona2008@126.com (X.-N.L.); xin.li@live.com (X.L.); shunliky@163.com (S.-L.W.); ldwswjsn1@126.com (D.-W.L.); machao3721@sina.com (C.-Y.M.); lixiaohui1978@163.com (X.-H.L.); 2 State Agriculture Biotechnology Centre, Western Australian Department of Agriculture and Food, Perth, WA 6150, Australia

**Keywords:** linear and nonlinear 2-DE, MALDI-TOF/TOF MS, comparative proteomics, drought stress, wheat grains

## Abstract

A comparative proteomic analysis of drought-responsive proteins during grain development of two wheat varieties Kauz (strong resistance to drought stress) and Janz (sensitive to drought stress) was performed by using linear and nonlinear 2-DE and MALDI-TOF mass spectrometry technologies. Results revealed that the nonlinear 2-DE had much higher resolution than the linear 2-DE. A total of 153 differentially expressed protein spots were detected by both 2-DE maps, of which 122 protein spots were identified by MALDI-TOF and MALDI-TOF/TOF mass spectrometry. The identified differential proteins were mainly involved in carbohydrate metabolism (26%), detoxification and defense (23%), and storage proteins (17%). Some key proteins demonstrated significantly different expression patterns between the two varieties. In particular, catalase isozyme 1, WD40 repeat protein, LEA and alpha-amylase inhibitors displayed an upregulated expression pattern in Kauz, whereas they were downregulated or unchanged in Janz. Small and large subunit ADP glucose pyrophosphorylase, ascorbate peroxidase and G beta-like protein were all downregulated under drought stress in Janz, but had no expression changes in Kauz. Sucrose synthase and triticin precursor showed an upregulated expression pattern under water deficits in both varieties, but their upregulation levels were much higher in Kauz than in Janz. These differentially expressed proteins could be related to the biochemical pathways for stronger drought resistance of Kauz.

## 1. Introduction

Drought stress is one of the major abiotic stresses in the world, which causes significant alterations in both yield and quality in many crop species during grain filling. It has been shown that drought stress during the crop flowering stage disrupts photosynthesis and transfer of stored carbohydrates into grains, which is the reason for the reduced grain number and weight [1]. The reduction was found to be more severe when the stress occurs at the early grain filling stages rather than at later stages [2]. Furthermore, remobilization of stored carbon reserves in wheat is promoted by water stress and water deficiency during grain filling, which enhances plant senescence and accelerates grain filling [3,4]. Thus, in order to improve wheat drought resistance and minimize yield loss, it is important to understand the mechanisms responsive to drought stress.

Wheat grain proteins are traditionally divided into prolamins and non-prolamins. The prolamins include gliadins and glutenins, while the non-prolamins consist of water-soluble albumins and salt-soluble globulins. The albumin and globulin proteins accumulate during the early stage of grain development, from flowering to 20 days after flowering (DAF), after which the content of those proteins remains stable. From flowering to 10–15 DAF, these proteins consist of mainly metabolic and structural proteins; but from 10–15 DAF onwards, the albumins and globulins start their accumulation in the developing starchy endosperm, consisting mainly of α,β-amylase/trypsin inhibitors and triticins [5–7]. It is generally accepted that wheat flour quality characteristics mainly depend on the composition and content of prolamins in the endosperm. In contrast, the role of albumins and globulins for the formation of flour quality is not as well defined as that of prolamins.

Albumins and globulins are mainly metabolic enzymes that participate in various metabolic activities during grain filling process, including protein synthesis, protein folding, starch synthesis and energy metabolism. Yield losses under drought stress are mainly caused by reduction in the starch content [8]. Starch accumulation is correlated with the sucrose content of kernels and with the activity of sucrose synthase and other enzymes related with starch synthesis [9]. Drought stress can affect the starch biosynthetic enzymes [10,11], as well as causing the accumulation of toxic metabolites. Some albumins and globulins have detoxification and defense functions that are associated with the resistance of plants to abiotic and biotic stresses. The albumins and globulins appear to play important roles in wheat yield and quality conformation under drought stress.

Protein synthesis and accumulation in wheat kernels have been characterized [12–14], and more recently, comparative proteomic analyses of wheat seeds, roots, leaves as well as embryos under drought and salt stresses have been carried out [15,16]. Relatively few reports have examined the changes of albumins and globulins in developing wheat grains under drought stress. In the present study, we performed a comparative proteomics analysis of drought-responsive proteins during grain development between two wheat varieties, Kauz and Janz, by using linear and nonlinear 2-DE and MALDI-TOF mass spectrometry technologies. The CIMMYT wheat variety Kauz shows a high concentration of shoot carbohydrates (*ca.* 40%) and is drought tolerant [17,18], while Janz is an Australia prime hard wheat cultivar which is drought sensitive [19,20]. Our results obtained here provide new insights into the drought-responsive mechanisms in wheat developing grains.

## 2. Results

### 2.1. Identification and Functional Classification of Drought-Responsive Proteins through Linear and Nonlinear 2-DE and MALDI-TOF Mass Spectrometry

**Figure 1:**
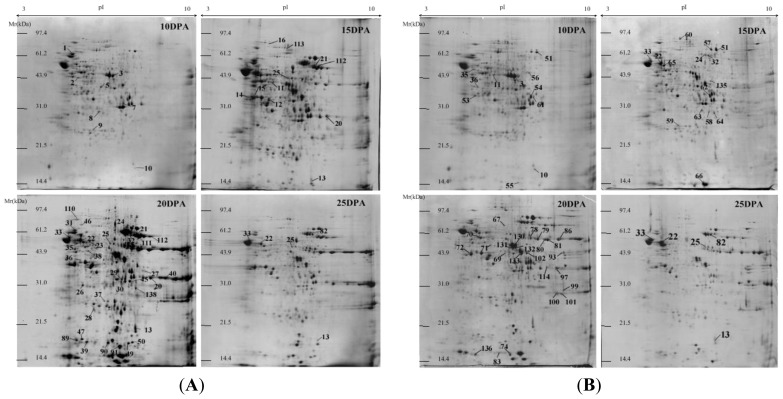
Proteome maps of wheat grain non-prolamins from four grain development stages (10, 15, 20 and 25 DPA) of two wheat varieties under drought stress by linear 2-DE (pH 3–10, 18 cm strips). (**A**): Janz, (**B**): Kauz. The numbered protein spots were identified by MALDI-TOF mass spectrometry and MALDI-TOF/TOF mass spectrometry. Differentially expressed proteins (>2 folds than the control) under drought stress are indicated in the gel.

**Figure 2:**
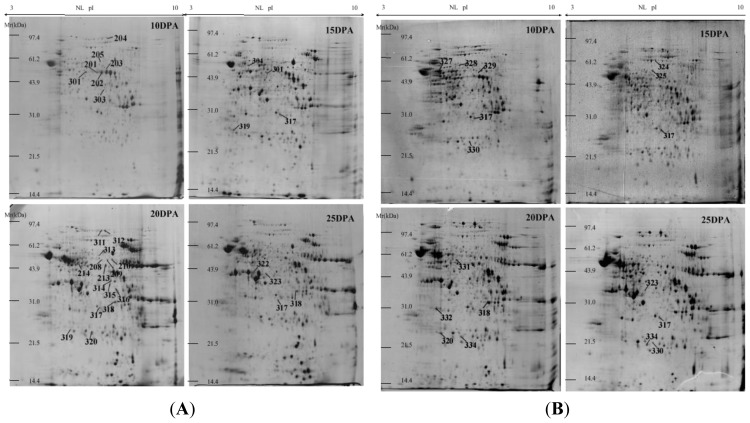
Proteome maps of wheat grain non-prolamins from four grain development stages (10, 15, 20 and 25 DPA) of two wheat varieties under drought stress by nonlinear 2-DE (pH 3–10, 18 cm strips). (**A**): Janz, (**B**): Kauz. Thirty-five newly identified differentially proteins (>2 folds than the control) under drought stress by nonlinear 2-DE and MALDI-TOF/TOF mass spectrometry were indicated in the 2D gel.

The linear (L) and nonlinear (NL) 2-DE results showed that molecular masses of protein spots ranged from 14 to 97 kDa and that most of them were in the pH 4–8 range under both electrophoretic methods. A total of 835 protein spots were separated by linear 2-DE gels, of which 417 spots could be matched across all gels (Figure 1A,B). However, when separated by nonlinear 2-DE, up to 1152 protein spots were identified and 573 of these were matched in all gels (Figure 2A,B). Thus, compared with linear 2-DE gels, the resolution of nonlinear 2-DE gels increased by 38%.

Although high similarity of protein profiles was observed, there was a significant difference in the expression patterns between the drought-stressed and the well-watered groups of both varieties. A total of 105 and 48 protein spots were detected as differentially expressed proteins by linear and nonlinear 2-DE, respectively, between the well-watered and drought stress treatments across the four grain developmental stages. Out of these 153 differentially expressed protein spots, 122 (79.7%) were identified successfully, including 24 spots by MALDI-TOF MS and 98 spots by MALDI-TOF/TOF MS. The identification results were listed in Supplementary Table S1 (by linear 2-DE) and Table S2 (by nonlinear 2-DE). The peptide sequences of identified proteins by tandem mass spectrometry were listed in Table S3.

**Figure 3:**
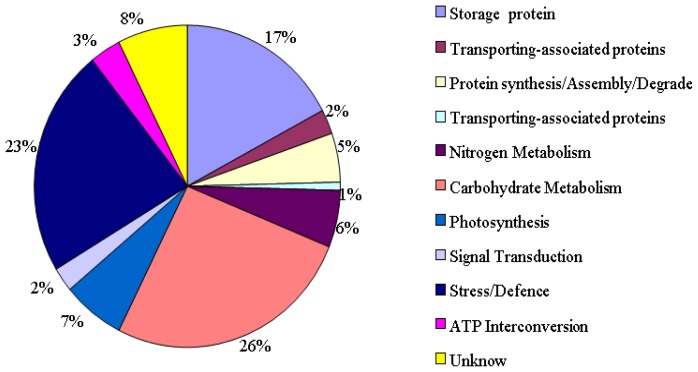
Functional distribution of 122 identified differentially expressed proteins during different grain development stages in Janz and Kauz under drought stress. A total of 11 functional catalogs and their percentages were shown.

Drought-responsive protein spots in both varieties were grouped into different functional categories. As shown in Figure 3, the 122 identified differential proteins were classified into 11 groups, which covered a wide range of molecular functions including carbon metabolism (26%), detoxification and defense (23%), storage proteins (17%), photosynthesis (7%), nitrogen metabolism (6%), protein synthesis/assembly/degrade (5%), ATP interconversion (3%), chaperones (2%), signal transduction-associated proteins (2%), translation associated proteins (1%), and unknown proteins (8%).

### 2.2. Protein-Expression Profiles under Well-Watered and Drought-Stress Conditions

**Figure 4:**
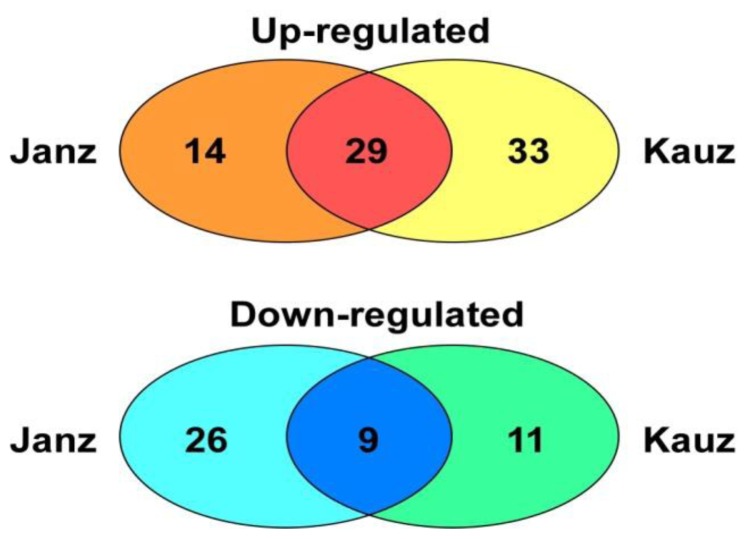
Venn diagram illustrating the expression comparison of drought-responsive proteins in developing grains between Janz and Kauz. The numbers indicated the proteins with up- and downregulated expression patterns during grain development in Janz and Kauz under drought stress.

By analyzing the accumulation patterns of differentially expressed proteins during grain filling, six main types of expression patterns under well-watered and drought-stress conditions were recognized (Figure 4). The pattern A contained 32 proteins and exhibited an upregulated expression such as serpin-Z2B (spot 15) that only had a trace expression at 10 days postanthesis (DPA) but rapidly upregulated from 10–25 DPA in Janz under both conditions. The B expression pattern including 35 proteins displayed a “Λ” type, including WD40 repeat protein (spot 26) in Kauz under drought stress and predicted r40c2 protein (spot 30) in Janz under well-watered conditions. The C pattern with eight proteins, contrary to the B pattern, presented a “V” type expression such as Rubisco (ribulose-1,5-bisphosphate carboxylase/oxygenase) large subunit (spots 201 and 202) in Kauz under drought stress. The D pattern displayed a “*N*”-shape expression, including nine proteins, such as predicted r40c2 protein (spot 30) in Kauz under well-watered conditions, beta amylase (spots 22 and 65) and dehydroascorbate reductase (spot 334) in Kauz, and sucrose synthase type 2 (spots 311 and 312) in Janz under drought stress. Contrary to the D pattern, the E expression had seven proteins and showed a “И” shape expression trend such as formate dehydrogenase (spot 69) in Kauz under drought stress. The F expression pattern was downregulated, including 31 proteins, e.g., HSP70 (spot 1) and predicted r40c2 protein (spot 30) in Kauz and Rubisco large subunit (spot 3) in Janz under drought stress.

In addition to the six main expression patterns, some protein spots only expressed in certain specific grain developing stages. For example, peroxidase 1 (spot 20) in Janz began to express at 15 DPA, and then displayed a “Λ” type expression trend under drought stress. Similarly, peroxidase 1 (spot 97) appeared at 15 DPA and continually increased in Kauz under drought stress. Chain A crystal structure of xylanase inhibitor protein (Xip-I) (spot 99) and basic endochitinase C (spot 100) only expressed in the last stage in Kauz under drought stress. Type I small heat shock protein 17.7 kDa I2I isoform (spot 47) expressed at 15 DPA in Janz while ATP-dependent RNA helicase eIF4A (spot 2) was only expressed in the first two stages in Janz under drought stress.

### 2.3. Comparative Proteomic Analysis between Janz and Kauz under Drought Stress

A comparative proteomics analysis of drought-responsive proteins between the two varieties during the grain developmental stages was performed, and significant expression differences were found. As shown in Figure 4, 29 protein spots (1, 3, 12, 14, 15, 20, 24, 30–32, 40, 45, 50, 51, 86, 97, 101, 111, 112, 133, 136, 138, 201–203, 205, 311, 312 and 320) were upregulated and nine protein spots (2, 8, 11, 35–37, 67, 317 and 318) were downregulated under drought stress in both varieties. In the Janz variety only, the expression abundances of 14 spots (7, 21, 28, 39, 46, 47, 49, 89–91, 214, 301, 303 and 314) increased, whereas 26 spots (5, 9, 10, 13, 16, 22, 23, 25–27, 33, 38, 110, 113, 208–210, 213, 304, 310, 313, 315, 316, 319, 322 and 323) displayed a downregulated expression under drought stress. Meanwhile, 33 protein spots (29, 54, 56–66, 72, 78–82, 99, 100, 102, 114, 130–132, 135, 204, 324, 325, 327, 330 and 334) showed an upregulated expression pattern while the expression of 11 spots (53, 55, 69, 70, 71, 74, 83, 328, 329, 331 and 332) had a downregulated pattern in Kauz only under drought stress.

Among the differentially expressed protein spots, 26% were involved in carbon metabolism. Of them, eleven spots in Janz (spots 5, 22, 25, 27, 33, 38, 208, 209, 304, 310 and 316), four spots in Kauz (spots 54, 70, 71 and 331) and one spot (12) in both varieties were downregulated under drought stress. However, there were still sixteen upregulated proteins. For example, under drought stress, glyceraldehyde-3-phosphate dehydrogenase (GAPDH, spots 7 and 21 in Janz, spots 54 and 62 in Kauz, spots 111 and 112 in both varieties) were upregulated at 20 DPA. Aldose reductase-related protein (spot 45), sucrose synthase type 2 (spots 311 and 312) and triosephosphat-isomerase (spot 320) were upregulated in both varieties under drought conditions. Spots 65 and 327 in Janz and spot 214 in Kauz that were identified as beta amylase were upregulated under drought stress.

**Figure 5:**
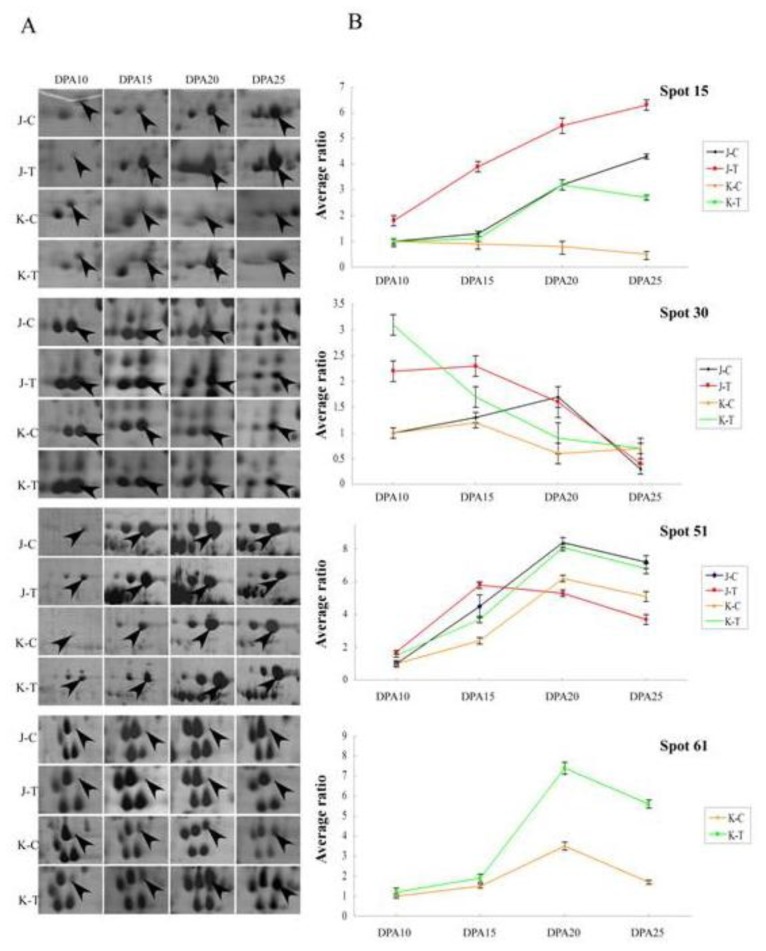
Expression patterns of four important drought-responsive proteins in Janz and Kauz under drought stress. A. The close-up view of the expression levels for four protein spots on 2-DE gels. B. The expression patterns of four protein spots. Average ratio: average ratio of the protein abundance (treated/control) on different drought concentrations. Spot 15: serpin-Z2B; spot 30: putative r40c2 protein; spot 51: putative oxygen-evolving enhancer protein 1; spot 61: triticin precursor. J: Janz, K: Kauz, C-Control, T-Treatment by drought stress.

Among the differentially expressed proteins, 24 belonged to the detoxification and defense group, in which eight protein spots (spots 9, 20, 37, 40, 56, 97, 114 and 319) were involved in antioxidant metabolism, and four spots (20, 40, 97 and 114) were identified as peroxidase 1. The intensity of peroxidase 1 (spots 20, 40 and 97) increased under water stress in both varieties, but spot 114 was upregulated only in Kauz. Three spots (9, 37 and 319) were identified as ascorbate peroxidase (APX), which displayed a downregulated expression pattern under drought stress in Janz and no changes in Kauz. Catalase isozyme 1 (spot 56) increased under water stress at 10 DPA in Kauz only. Two isoforms of OSR40 family (spot 29 and 30) were identified and putative r40c2 protein (spot 30) was obviously upregulated at 10 DPA under drought stress in Kauz. Its expression was gradually reduced during grain development with its expression level higher in drought condition than the controls (Figure 5). In Janz, its expression was slightly upregulated under drought stress in the first two stages, and then it remained stable without any difference between the two treatment groups (Figure 5).

Ten spots (14, 15, 66, 72, 99, 136, 138, 314, 322 and 323) were identified belonging to protease inhibitor family. In particular, alpha-amylase/trypsin inhibitor CM3 (spot 66), Chain A, crystal structure of xylanase inhibitor protein (spot 99), 0.19 dimeric alpha-amylase inhibitor (spot 136) and endogenous alpha-amylase/subtilisin inhibitor (spot 138) were upregulated in Kauz under drought stress. In Janz, alpha-amylase/trypsin inhibitor CM3 (spot 314) was downregulated, but its isoform spot (138) was upregulated. Five protein spots were identified as serpin in this family and displayed a different expression pattern between the two varieties. For example, spot 15 gradually accumulated from 10 to 25 DPA in Janz under both treatments with the drought stress group possessed a higher abundance than the control (Figure 5). Whereas in Kauz, its expression level was lower in the control group and increased rapidly from 15 to 20 DPA in the drought stress group (Figure 5). Three spots (spots 16, 60 and 113) were identified as late embryogenesis abundant (LEA) protein, of which spot 16 and 113 showed downregulated in Janz and spot 60 displayed an upregulated expression pattern in Kauz under drought stress.

Only three differentially expressed protein spots were identified as signal transduction proteins, including G beta-like protein (spot 8), WD40 repeat protein (spot 26) and Os04g0118400 (spot 204). The G beta-like protein was downregulated in Janz, but the WD40 repeat protein and Os04g0118400 were both upregulated in Kauz under drought stress.

Three protein spots (3, 201 and 202) were identified as the Rubisco large subunit belonging to the photosynthesis group, which displayed upregulated expression patterns in both varieties under drought stress. Oxygen-evolving enhancer protein (OEE) 1 (spot 51) showed increased expression in Kauz under drought stress across all development stages; whereas in Janz at the first two stages, its expression was higher in the drought stress group compared with the control group, but at the last two stages its expression became higher in the control group (Figure 5). The 23kDa polypeptide of photosystem II (spot 28) was upregulated in Janz under drought stress. The protein synthesis, assembly and degrade-related proteins, represented by six protein spots (13, 47, 53, 58, 59 and 110), were also found to be differentially expressed. Under drought stress, sequence 5 from patent US-5668007 (spot 13), annexin 2 (spot 58) and putative proteasome subunit alpha type 3 (spot 59) were upregulated in Kauz; type I small heat shock protein 17.7 kDa I2I isoform (spot 47) and cytosolic heat shock protein 90 (spot 110) were downregulated in Janz. Interestingly, 21 differentially expressed spots were identified as storage proteins and were generally showing upregulation in drought conditions. Under drought stress, triticin precursor spots (61, 101, 130–133) were all increased in Kauz, but only spots 101 and 133 were increased in Janz. Among them, spot 61 was only found in Kauz and its expression was increased dramatically from 15 DPA under drought stress (Figure 5). The chaperone protein HSP70 (spots 1 and 31) was upregulated at 10 DPA in both varieties, but was downregulated at 20 DPA in Janz under drought stress. Cyclophilin-like protein (spot 10) was downregulated at 10 DPA in Janz under drought stress. In terms of the nitrogen metabolism related proteins, NAD-dependent formate dehydrogenase (spot 69) showed a downregulated pattern in Kauz under drought stress. Spot 102 and 135 were identified as unnamed protein products with putative similar function to Ketol-acid reductoisomerase, which showed an upregulated expression in Kauz under drought stress.

## 3. Discussion

In the current study, we described the dynamic changes of wheat drought-responsive proteins during grain development and conducted a comparative proteomics analysis between two varieties that differ widely in drought tolerance by using linear and nonlinear 2-DE and MALDI-TOF MS approaches. Since plants have the ability to synthesize series of functional proteins to fight drought stress, our results provide new insights into proteomic mechanisms of wheat drought tolerance during developing grains.

### 3.1. Linear and Nonlinear 2-DE for Wheat Protein Maps

Common wheat has a large genome size, being about 35 times larger than that of rice and about 110 times that of Arabidopsis [21]. The wheat genome project has not been completed yet, which has resulted in a considerable difficulty for wheat proteome analysis due to lack of data directly derived from wheat across various databases. Compared to model crop rice and other plants with small genome sizes, high resolution 2-DE method is highly important for the wheat proteome map. In recent years, linear strips (pH 3–10) have been widely used for wheat seed proteome separation, but limited number of protein spots was resolved, being generally less than 800 protein spots [12]. In this study, we conducted a comparative analysis between linear and nonlinear 2-DE analysis and found that the resolution of nonlinear 2-DE was much higher than that of linear 2-DE. Since the isoelectric point distributions of linear and nonlinear strips are even and uneven, respectively, the middle pH range (pH 5–7) in nonlinear pH 3–10 strips has a wider distribution on the strip than that on linear strips. Our results indicated that a great number of albumins and globulins in wheat grains located in this middle pI range on 2-DE gels (Figures 1 and 2), and therefore a higher resolution was obtained by using nonlinear strips. This suggests that nonlinear 2-DE is more suitable for wheat proteome maps.

### 3.2. Detoxification and Defense Proteins

Reactive oxygen species, such as singlet oxygen, superoxide radical, hydroxyl radical and hydrogen peroxide, often accumulate in plant cells under drought stress [22]. Catalase, APX and peroxidase are enzymes that catalyze the conversion of H_2_O_2_ to H_2_O and O_2_. Our results showed that the expression intensity of peroxidase 1 increased under drought in both varieties; the same phenomenon was present in the root and needle of Norway spruce [23] and leaves of *Ramonda serbica*[24]. The activity of peroxidase was upregulated under drought, which may be considered as part of an antioxidative defense. Previous reports indicated that cytosolic isozymes of APX and catalase showed an increased expression in pea shoots [25], but the activity of APX remained unchanged in leaves of *Ramonda serbica* during drought stress [24]. In the present work, the expression of APX decreased in Janz under drought stress, but had no change in Kauz, suggesting that Kauz is more drought resistant than Janz. Catalase activities was reported to increase or be stable in the early stage of drought stress, and then decrease with further increase in magnitude of water stress [26]. Our results showed that catalase isozyme 1 only increased at 10 DPA under drought stress in Kauz. These indicated that catalase might be activated to scavenge toxic compounds during an early stage when the plant adapts the drought stress.

Protease inhibitors generally express in a constitutive manner in many plants, particularly in storage tissues such as seeds and tubers after induction by a biotic stress [27–29]. Alpha-amylase inhibitors may offer protection against oxidative stress and help preserve grain starch, a component crucial for germination [30]; they also have important roles in resisting insect damage in plants [31]. They displayed an upregulation of individual forms in tolerant Khazar-1 and downregulation in the susceptible genotypes [32]. Our results also showed that two alpha-amylase inhibitors were upregulated in Kauz and one downregulated in Janz, which may account for the higher drought tolerance and starch accumulation of Kauz.

Serpin is one of the two peptidase inhibitor families found in all domains of life [33]. It is reported that serpin accounted for several percent of the total proteins in wheat grains [34]. The abundance of seed serpin (possibly present in other organs/tissues) is likely to be involved in direct defense or related to the regulation of defense activation and/or programmed cell death [35]. Two serpin spots (322 and 323) were down regulated in Janz under drought stress. This may partly explain the low drought tolerance of Janz.

The salt-stress-responsive plant gene family OSR40 that encodes hydrophilic proteins with duplicated domains was characterized in a salt-tolerant rice variety and was found to be induced by saline stress and ABA [36,37]. Structural proteins such as OSR40 could help to prevent water loss and preserve the rigidity of cells thus play an important role in response to osmotic stress [37]. A BLAST search against *Oryza sativa* by using OSR40 (gi|34902150) as query returns six significant subjects: r40c1, r40g2, r40c2, r40g3, an unknown protein, and a putative r40c1 [38]. In our results, the predicted r40c2 protein was found and showed a significant increase compared with the control in responding to drought stress at 10 DPA in both varieties, which is in agreement with a previous report that a protein similar to rice OSR40 showed an upregulation under water deficiency in maize leaves [39].

### 3.3. Carbohydrate Metabolism Proteins

Carbon metabolism proteins generally demonstrate a great change under drought stress. Studies showed that carbon metabolism enzymes are accumulated during grain filling stages such as sucrose synthase, starch synthase and other enzymes which can provide cofactors not only for the synthesis of protein and lipids, but also for CO_2_ refixed [40]. The grain filling is actually the process of starch biosynthesis and accumulation in endosperm cells. The synthesis of starch mainly occurred in amyloplast, catalysed by series of enzymes such as ADP-glucose pyrophosphorylase, starch synthase and starch debranching enzyme [41]. ADP-glucose pyrophosphorylase (AGPase) is regarded as rate-limiting enzyme in starch biosynthesis. It catalyzes the first step of starch biosynthesis by generating the sugar nucleotide ADP-glucose and inorganic pyrophosphate (PPi) from glucose-1-phosphate and ATP. Grain yield and plant biomass increases are conferred by deregulation of endosperm AGPase [42]. The AGP expression was showed to be inhibited by water deficiency in wheat microspore mother cells [10]. Our results demonstrated that small and large subunit AGPase were both downregulated under drought stress in Janz, but had little change in Kauz, suggesting that Janz is sensitive to drought stress.

Sucrose synthase can not only catalyse the process of sucrose decompose into fructose and UDP-glucose, but also catalyze the reversible reaction [43]. In the cytoplasm of grain endosperm cells, sucrose is constantly split to UDP-glucose used for the synthesis of starch and other carbohydrates [44]. In sink tissues, the degradation of sucrose was mainly catalyzed by sucrose synthase [45]. Our results are well in agreement with the report of Saeedipour and Moradi [11], in which the sucrose synthase activities in grains were higher under water deficiency in both varieties with obvious further activity in the drought-tolerant variety. The rise in sucrose synthase activity is positively correlated with the onset of starch and storage protein biosynthesis [46]. We speculated that the increase of sucrose synthase activity under drought stress may enhance the osmotic adjustment ability of plants and could provide more material for glycolysis, and consequently result in more starch and storage proteins. This correlates with the higher drought tolerance of Kauz.

Aldose reductase (ALR) is a cytosolic monomeric enzyme with broad substrate specificity, ranging from sugars to aromatic aldehydes [47]. It is a rate-limiting enzyme in the polyol pathway associated with the conversion of glucose to sorbitol. The ALR gene in plants expressed constitutively during embryo maturation and is modulated by plant hormones ABA and gibberellic acid [48]. It was postulated that *Avena fatua* ALR may play an important metabolic role in desiccation tolerance [49]. Furthermore, salt stress increased the activity of ALR in NaCl-stressed seedlings of foxtail millet [50]. ABA and osmotic stress induce the expression of genes encoding ALR [48]. In our study, drought stress increased the expression of ALR and we speculate that this may increase the intracellular sorbitol which was believed to maintain the osmotic balance and cell volume.

### 3.4. Signal Transduction Related Proteins

Signal transduction in drought stress consists of ionic and osmotic homeostasis signaling pathways, detoxification (*i.e.*, damage control and repair) response pathways, and growth regulation pathways [51]. The G-β subunit containing seven repeats of WD40 motif, is the best characterized WD40 protein [52]. Structurally, these proteins contain a conserved Trp-Asp motif (the so-called WD40 repeat), a ~40-amino acid stretch typically ending in Trp-Asp, but exhibiting only limited amino acid sequence conservation [53]. These WD40 proteins may play key roles in signal transduction, cytoskeletal dynamics, protein trafficking, nuclear export, ribosomal RNA biogenesis and especially in chromatin modification and transcription [54,55]. Horsegram (*Macrotyloma uniflorum* (Lam.) Verdc.) is a legume crop that can tolerate severe adverse environmental conditions such as drought, salinity and heavy metal contamination; it can also transcript WD40 repeat family proteins under drought stress [55]. All five *SRWD* genes encoding a novel protein with five WD40 repeats in rice were regulated by salt stress [56]. In our study, WD40 repeat protein showed an upregulated expression in Kauz, but had no change in Janz under drought stress, identifying this protein as a potential candidate for enhancing plant drought tolerance.

Guanine nucleotide binding protein (G proteins) is a family of proteins involved in second messenger cascades. G proteins are thus called because of their function of “molecular switches,” alternating between an inactive guanosine diphosphate (GDP) and active guanosine triphosphate (GTP) bound state, ultimately going on to regulate downstream cell processes. G proteins belong to a family of WD-repeat proteins serving as modulators or transducers in various transmembrane signaling systems [57]. The activity of phospholipase C in plants may be regulated by G-proteins, and phosphoinositols modulate the expression of these LEA-like genes under cold, drought and salt stress. G-protein-associated receptors may exist and function in the perception of a secondary signal derived from these stresses [58]. The phosphoinositide signal transduction pathway is of major importance in the plant response to salinity and drought stresses [59]. Our results showed that G beta-like protein was downregulated in Janz under drought stress, but had no change in Kauz, thereby correlating with the fact that Janz is a drought-sensitive variety.

### 3.5. Photosynthetic Proteins

The seeds of many plant species are green during their early development; some of them can use weak light for photosynthesis. Tschiersch *et al.* localized and described gradient distributions of photosynthetic activity across the seed/caryopsis, and discussed its role in maintaining the endogenous O_2_ balance [60]. They also speculated that the photosynthesis of seeds was related with salt and drought environment.

Oxygen evolving enhancer proteins consist of three subunits, OEE1 (33 kDa), OEE 2 (23 kDa) and OEE 3 (16 kDa). The N-terminus of the 33 kDa protein is necessary for maintaining the binding ability of the protein to Photosystem II, but might not be involved in binding itself [61]. OEE1 has a protective effect on the manganese cluster, also known as the manganese stabilizing protein. The expression of OEE1 is also considered to be the rate-limiting step in the assembly of PSII subunit [62]. The expression of OEE1 increased under NaCl treatment, and salt treatment can improve the level of gene transcription in the mangrove *Bruguiera gymnorrhiza*[63]. Water deficiency also increases the activity of OEE1 in maize leaves [39]. In our study, water deficiency increased the expression of OEE1 in both varieties at the first two stages; however, under water stress, its expression was upregulated in Kauz, but downregulated in Janz at the last two stages. Therefore, we speculate that the increase of OEE1 is one of the mechanisms to maintain the capacity of PSII under drought, thereby making Kauz more drought tolerant than Janz.

### 3.6. Storage Proteins

Certain albumins and globulins are considered to have storage protein functions in wheat seeds. For example, the content of triticins at maturity accounts for about 5% of the total grain proteins [5], and particularly high molecular weight albumin subunits (mainly β-amylases) account for 8%–10% of the polymeric protein fractions [7]. In our experiment, embryo globulin, globulin 3B, globulin 3, triticin precursor and avenin-like protein were identified as storage proteins and all displayed upregulated expression during grain development under drought stress, which could benefit wheat quality conformation. A previous report also demonstrated that drought stress could elevate the quantity of grain proteins, which would be useful for improving flour quantity [64]. On the other hand, wheat albumins and globulins are rich in lysine, tryptophan and methionine and have well-balanced amino acid compositions, and thus have comparatively high nutrient values [65]. The upregulation expression of these storage proteins under drought stress could improve grain nutritional quality.

Osmotic stress under drought environment is the main cause of reduced plant yield and even death. The synthesis of osmotic regulation substances such as amino acids, sugar alcohols and other water-soluble molecules is an important way for plants to resist drought stress. Studies showed that, fructan as a soluble carbohydrate, resolves to produce monosaccharide, which can improve the vacuole juice of sugar concentration, decrease osmotic pressure, thereby enhancing the ability of plant cells against stress [66]. Some investigations demonstrated that transferring the SacB gene into plants that cannot produce fructan can make these plants acquire the ability to accumulate fructan, and thus improve the tolerance of these plants [67,68]. In our work, all triticin precursor spots were increased under drought stress in Kauz, but only two spots increased in Janz.

Avenin-like proteins were found in wheat endosperm in recent years, which were similar to the oat storage proteins. They usually contained 18 to 19 cysteine residues, which is much more than that in all other glutenin subunits characterized so far. Thus, they were predicted to form several disulfide bonds [69]. Gliadins mainly form intramolecular disulfide bonds, which lead to its existence as monomeric proteins and imparts viscous property to dough [70]. In contrast, glutenins are polymeric, in which the constituent polypeptides (subunits) are linked by interchain disulfide bonds and confer viscoelastic property to dough [71]. The presence in the glutenin extract and the high number of cysteine residues suggested that avenin-like protein type-B is through interchain disulfide bonds integrated into gluten polymers [72]. The cysteine residues in avenin-like proteins may form intramolecular or interchain disulfide bonds, and therefore they may have a significant effect on wheat-processing quality. Similar to the other storage proteins in this work, the expression of avenin-like proteins showed to be upregulated under drought stress in Kauz, but no significant change in Janz. This suggests that drought-tolerant varieties may have better quality, as well as higher yield than drought-sensitive varieties under water deficiency.

### 3.7. A Putative Basis of Drought Response and Tolerance in Respect to Wheat Grain Development

Wheat grain development is a complex process, which is highly related to grain yield and quality. Particularly, wheat storage starch, a major component of yield, accounts for two thirds to three-quarters of the final grain weight [73]. In general, wheat needs a lot of water for starch and protein synthesis during grain filling. When plants encounter drought stress at this stage, the regular expression of many proteins related to stress/defense/detoxification, carbohydrate metabolism, photosynthesis and nitrogen metabolism are disturbed, leading to a severe yield loss. However, plants can develop complex signaling networks to adapt to harsh environments. In the current study, a number of proteins were identified to be putatively important for fighting the water deficiency. Under drought stress, these proteins, including APX, alpha-amylase inhibitors, serpin, small and large subunit AGPase, G beta-like protein and OEE1 that are related to various metabolic processes, displayed significant downregulations in the drought-susceptible variety Janz and upregulated expressions in the drought-resistant variety Kauz. It could be concluded that these proteins, as well as some storage proteins, may be responsible for the two varieties’ differences in response to water deficiency.

## 4. Experimental Section

### 4.1. Plant Materials, Treatments and Sample Collection

Two wheat varieties (*Triticum aestivum* L., 2*n* = 6*x* = 42, AABBDD), including an Australian cultivar Janz and a CIMMYT wheat variety Kauz, were grown in a glasshouse at the Chinese Academy of Agricultural Sciences (CAAS), Beijing, from September 2010 to February 2011. Six seeds were grown in a 20 cm (D) × 25 cm (H) pot with mixed soil (black soil:peat = 2:1). The glasshouse temperatures ranged from 20 to 25 °C, and the humidity was maintained between 60% and 70%. After heading, half of the pots were restricted with watering (water deficiency treatments, while the other half of the pots were kept well watered until maturity. The soil water contents of two treatment groups were measured and the drought stress group was maintained at about one third soil water content of the well-watered control. Grain samples from middle spike were collected from four developmental stages, including 10 (I), 15 (II), 20 (III) and 25 (IV) days postanthesis (DPA) with three replicates. The collected grains were stored at −80 °C prior to analysis.

### 4.2. Protein Extraction and Two-Dimensional Electrophoresis

Grain albumin and globulin proteins from the collected samples were extracted according to Gao *et al.*[12]. Protein separation was performed by both linear and nonlinear two-dimensional electrophoresis (2-DE). Protein concentration for each sample was measured by 2-D Quant Kit (GE Healthcare, Piscataway, NJ, USA) and 600 μg proteins were loaded onto analytical and preparative gels. Ettan IPGphor system and pH 3–10 IPG strips (18 cm, Linear and Nonlinear, GE Healthcare, Piscataway, NJ, USA) were used for the isoelectric focusing (IEF). The IEF rehydration solution was 7 M urea, 2 M thiourea and 4% CHAPS. The rehydrate condition was 30 V at 20 °C for 12 h while the IEF condition was 300 V for 1 h 500 V for 1 h, 1000 V for 1 h, 3000 V for 1 h, and 8000 V to 80,000 V·h. SDS-PAGE was performed using 12% gels.

### 4.3. Gel Scanning and Image Analysis

After electrophoresis, the 2-DE gels were stained with colloidal Coomassie Brilliant blue (CBB) (R-250/G-250 = 4:1) and analyzed by using ImageMaster™ 2-D platinum software version 5.0 (Amersham Bioscience, Swiss Institute of Bioinformatics, Geneva, Switzerland, 2003). The spots in 2-DE gels were quantified using the percent volume criterion, and the relative volume was considered as its expression level. Automatic match analysis was performed, and then the mismatched and unmatched spots were corrected by manual editing. Protein spots that showed statistically significant changes between samples were determined by Student’s *t*-test (abundance variation at least 2-fold, *p* < 0.05). After image analysis, protein spots showing significant changes in abundances were selected for further MS analysis.

### 4.4. Proteolytic Digestion and MS Analysis

Differentially expressed proteins were cut from gels and soaked in decoloring liquid until the whole gel was colorless. After the decoloring liquid was discarded, 200 μL acetonitrile (ACN) was added until the samples turned white and the sample was then dried for 30 min using a vacuum dryer. The dried samples resuspended in 20 μL enzyme solution to be hydrolyzed for 16 h at 37 °C. After digestion, the peptides were extracted once with 5% TFA (trifluoroacetic acid) and twice with 2.5% TFA. The supernatant containing peptides was thoroughly dried in a vacuum dryer and the dried peptide mixture was completely dissolved in 2 μL solution containing 5 mg/mL α-cynao-4-hydroxycinnamic-acid (CHCA, Sigma, Germany) in 50% ACN and 0.1% TFA.

Tryptic peptides were analyzed with MALDI-TOF mass spectrometer (SM, Shimadzu Biotech, Kyoto, Japan) and MALDI-TOF/TOF mass spectrometer 4800 Proteomics Analyzer (Applied Biosystems, Framingham, MA, USA). All MS and MS/MS spectra were searched in the NCBI nonredundant green plant database. The peptide mass tolerance and fragment mass tolerance were set to 0.2 Da and the MS/MS tolerance 0.3 Da. One missed cleavage variable modification of carbamidomethyl (Cys) and oxidation (Met) was specified as variable modification. GPS Explorer™ software version 3.6 (Applied Biosystem, Foster City, CA, USA) and Mascot 2.2 were used to get peptides sequence information. All proteins identified have the MASCOT total ion scores greater than 42 and the identification probabilities were more than 95%.

## 5. Conclusions

Our results demonstrated that the 2-DE resolution could be significantly imorived by the nonlinear (NL) strips compared to the linear ones, indicating that NL 2-DE is a more powerful tool for studies on wheat grain proteome. Comparative proteomic analysis between drought-resistant and sensitive wheat varieties Kauz and Janz during grain development under drought stress revealed 153 differentially expressed protein spots, of which 122 protein spots were identified by MALDI-TOF and MALDI-TOF/TOF mass spectrometry. These proteins were mainly involved in carbohydrate metabolism (26%), detoxification and defense (23%), and storage proteins (17%). Some key proteins, including catalase isozyme 1, WD40 repeat protein, LEA, alpha-amylase inhibitors, small and large subunit ADP glucose pyrophosphorylase, ascorbate peroxidase, G beta-like protein, sucrose synthase and triticin precursor displayed significantly different expression patterns in two varieties under drought stress, which could be related to stronger drought resistance of Kauz.

## Supplementary Information
