# Supplementary material for: Wheat Drought-Responsive Grain Proteome Analysis by Linear and Nonlinear 2-DE and MALDI-TOF Mass Spectrometry
Source: Int J Mol Sci. 2012 Nov 29;13(12):16065–83. doi: 10.3390/ijms131216065 (PMC3546679; doi:10.3390/ijms131216065)
Supplement: Supplementary file 1 [file ijms-13-16065-s001.pdf]

Supplementary Information

Table S1. Differentially expressed non-prolamins under control and drought stress conditions in Janz and Kauz by linear 2-DE (pH 3–10, 18 cm) and MALDI mass spectrometry.

Sport ID	Protein name	Plant species	Accession No (gi:)	Theor. Mr/pI [a]	Exp. Mr/pI [b]	Score [c]	PN [d]	Method [e]
**carbon metabolism**								
5	isocitrate dehydrogenase NADP-dependent	*Medicago truncatula*	92875135	48/6.02	45/5.99	98	14	MS
22	beta amylase	*Triticum aestivum*	32400764	31.1/8.6	60/5.42	312	9	MS/MS
33	beta amylase	*Triticum aestivum*	32400764	31.1/8.6	62/5.16	389	8	MS/MS
65	beta amylase	*Triticum aestivum*	32400764	31.1/8.6	59/5.53	573	13	MS/MS
111	Glyceraldehyde-3-phosphate dehydrogenase	*Triticum aestivum*	120680	36/6.67	78/6.25	416	14	MS
112	Glyceraldehyde-3-phosphate dehydrogenase	*Triticum aestivum*	148508784	36/7.08	78/6.37	597	17	MS
7	cytosolic glyceraldehyde-3-phosphate dehydrogenase	*Triticum aestivum*	7579064	25.4/7.83	40/6.65	348	9	MS/MS
21	Glyceraldehyde 3-phosphate dehydrogenase	*Triticum aestivum*	120680	36.0/6.67	65.5/6.65	416	14	MS
54	Glyceraldehyde 3-phosphate dehydrogenase	*Triticum aestivum*	18978	42/6.89	36/6.67	94	12	MS
62	Glyceraldehyde 3-phosphate dehydrogenase	*Triticum aestivum*	18978	42/6.82	36/6.67	351	4	MS
12	small subunit ADP glucose pyrophosphorylase	*Triticum aestivum*	7340287	52/5.53	44/5.74	216	12	MS
38	small subunit ADP glucose pyrophosphorylase	*Triticum aestivum*	7340287	52.3/5.53	53/5.61	761	21	MS/MS
25	ADP-glucose pyrophosphorylase large subunit	*Triticum aestivum*	995746	6.19/57	6.12/57	87	14	MS

Table S1. *Cont.*

27	cytoplasmic aldolase	*Oryza sativa Japonica Group*	218157	39.2/6.56	41/7.05	397	11	MS/MS
70	Xylose isomerase	*Triticum aestivum*	6175480	53.9/5.31	56/5.50	397	16	MS/MS
71	NADPH producing dehydrogenase of the oxidative pentose phosphate pathway	*Zea mays*	162463282	53.3/5.92	51/5.81	411	14	MS/MS
63	glucose and ribitol dehydrogenase homolog - barley	*Oryza sativa Japonica Group*	7431022	31.9/6.54	35/6.51	201	7	MS/MS
45	aldose reductase-related protein	*Bromus inermis*	167113	36.1/6.28	37/6.7	309	11	MS/MS
67	Os01g0743500	*Oryza sativa Japonica Group*	115439879	64.6/6.5	65/6.15	141	8	MS/MS
**Detoxification and defense**								
20	peroxidase 1	*Triticum aestivum*	22001285	39.3/8.14	38/7.25	416	13	MS/MS
40	peroxidase 1	*Triticum aestivum*	22001285	39.3/8.14	38/7.67	235	13	MS/MS
97	peroxidase 1	*Triticum aestivum*	22001285	39.3/8.14	38/7.5	333	16	MS/MS
114	peroxidase 1	*Triticum aestivum*	22001285	39.3/8.14	39/7.23	228	12	MS/MS
35	polyphenol oxidase	*Triticum aestivum*	46946550	46.9/5.23	57/5.32	440	17	MS/MS
14	Serpin-Z2B	*Triticum aestivum*	75279909	43.0/5.18	45/5.11	862	15	MS/MS
15	Serpin-Z2B	*Triticum aestivum*	75279909	43/5.18	45/5.26	624	14	MS/MS
72	Serpin-Z2B	*Triticum aestivum*	75279909	43.0/5.18	45/5.31	235	13	MS/MS
56	Catalase isozyme 1	*Hordeum vulgare*	1705612	57.1/6.68	54/6.72	360	15	MS/MS
66	Alpha-amylase/trypsin inhibitor CM3	*Triticum aestivum*	123957	18.9/7.44	14/6.56	512	7	MS/MS
136	0.19 dimeric alpha-amylase inhibitor	*Triticum aestivum*	56480630	13.8/5.23	13/5.43	620	9	MS/MS

Table S1. *Cont.*

138	Endogenous alpha-amylase/ subtilisin inhibitor	*Tricum aestivum*	12397	19.8/6.77	34/6.86	823	19	MS/MS
55	Os05g0453700	*Oryza sativa Japonica Group*	115464233	18.1/6.29	18/6.41	79	5	MS/MS
29	Os07g0683900	*Oryza sativa Japonica Group*	115474137	39.0/6.81	38/6.51	141	6	MS/MS
9	Ascorbate peroxidase	*Triticum aestivum*	3688398	30/5.84	27/5.85	91	11	MS
37	Ascorbate peroxidase	*Triticum aestivum*	15808779	29/6.04	27/5.15	103	2	MS
16	Group 3 late embryogenesis abunelate protein	*Triticum aestivum*	170692	33/5.01	77/6.10	164	4	MS
60	Group 3 late embryogenesis abunelate protein (LEA)	*Triticum aestivum*	21637	82/6.19	23/8.81	74	3	MS
113	LEA protein	*Triticum turgidum subsp. Durum*	48093951	77/6.17	22/9.22	135	5	MS
30	putative r40c2 protein	*Oryza sativa Japonica Group*	34394518	32.3/6.89	38/6.58	213	7	MS/MS
46	BAC19.13	*Solanum lycopersicum*	9858781	59.7/4.98	66/5.59	76	4	MS/MS
100	Basic endochitinase C	*Secale cereale*	75262903	28.7/8.82	28/7.51	296	6	MS/MS
99	Chain A, Crystal Structure Of Xylanase Inhibitor Protein (Xip-I) From Wheat	*Triticum aestivum*	31615809	30.5/8.27	29/7.67	407	13	MS/MS
**Storage protein**								
24	embryo globulin	*Hordeum vulgare subsp. vulgare*	167004	72.6/6.8	65/6.58	198	16	MS/MS
32	embryo globulin	*Hordeum vulgare subsp. vulgare*	167004	72.6/6.8	66/6.67	237	15	MS/MS
57	embryo globulin	*Hordeum vulgare subsp. vulgare*	167004	72.6/6.8	72/6.81	189	10	MS
78	embryo globulin	*Hordeum vulgare subsp. vulgare*	167004	72.6/6.8	56/6.83	173	13	MS/MS
79	embryo globulin	*Hordeum vulgare subsp. vulgare*	167004	72.6/6.8	55/7.02	234	15	MS/MS
80	embryo globulin	*Hordeum vulgare subsp. vulgare*	167004	72.6/6.8	55/7.21	202	12	MS/MS
81	embryo globulin	*Hordeum vulgare subsp. vulgare*	167004	72.6/6.8	54/7.27	154	11	MS/MS
82	embryo globulin	*Hordeum vulgare subsp. vulgare*	167004	72.6/6.8	67/6.77	196	12	MS/MS
86	embryo globulin	*Hordeum vulgare subsp. vulgare*	167004	72.6/6.8	54/7.54	218	13	MS/MS

Table S1. *Cont.*

61	triticin precursor	*Triticum aestivum*	7548844	57.3/9.37	42/6.87	88	7	MS/MS
101	triticin precursor	*triticin precursor*	7548844	57.3/9.37	28/7.61	624	12	MS/MS
130	triticin precursor	*Triticum aestivum*	7548844	57.3/9.37	51/6.46	759	14	MS/MS
131	triticin precursor	*Triticum aestivum*	7548844	57.3/9.37	49/6.37	821	15	MS/MS
132	triticin precursor	*Triticum aestivum*	7548844	57.3/9.37	49/6.46	823	17	MS/MS
133	triticin precursor	*Triticum aestivum*	7548844	57.3/9.37	46/6.60	510	12	MS/MS
39	globulin 3B	*Triticum aestivum*	215398472	57.1/7.36	18/5.57	139	8	MS/MS
49	globulin 3	*Triticum aestivum*	215398470	66.7/7.78	18/6.66	330	9	MS/MS
50	globulin 3	*Triticum aestivum*	215398470	66.7/7.78	18/6.78	312	8	MS/MS
89	globulin 3	*Triticum aestivum*	215398470	66.7/7.78	19/5.28	368	11	MS/MS
90	globulin 3	*Triticum aestivum*	215398470	66.7/7.78	18/6.14	218	10	MS/MS
64	avenin-like protein	*Triticum aestivum*	145321072	33.7/8.29	33/6.83	324	5	MS/MS
**Photosynthesis**								
28	23kDa polypeptide of photosystem II	*Oryza sativa Japonica Group*	2570499	27.2/9.06	26/5.78	85	6	MS/MS
3	Rubulose-1,5-bisphosphate carboxylase/oxygenase large subunit	*Psathyrostachys huashanica*	51859667	53/6.35	52/6.13	128	17	MS
51	putative oxygen-evolving enhancer protein 1	*Oryza sativa Japonica Group*	109892873	37/4.8	36/8.80	100	2	MS
83	Rubulose-1,5-bisphosphate carboxylase/oxygenase small subunit	*Triticum aestivum*	11990893	15/6.01	19/9.06	110	12	MS
**Nitrogen Metabolism**								
69	NAD-dependent formate dehydrogenase	*Hordeum vulgare*	21263612	41.7/6.9	46/6.18	286	11	MS/MS
102	Os01g0760600	*Oryza sativa Japonica Group*	115440075	50.1/8.61	46/6.71	350	10	MS/MS

Table S1. *Cont.*

135	Os01g0760600	*Oryza sativa Japonica Group*	115440075	50.7/8.61	49/6.72	188	9	MS/MS
23	Os05g0573700	*Oryza sativa Japonica Group*	115465569	62.7/6.01	59/5.67	291	9	MS/MS
**Protein synthesis/Assembly/Degrade**								
53	glutamine synthetase isoform GSr1	*Triticum aestivum*	40317416	39.0/5.35	44/5.36	457	12	MS/MS
59	putative proteasome subunit alpha type 3	*Oryza sativa Japonica Group*	50080306	27.5/5.76	29/5.90	238	10	MS/MS
58	annexin2	*Zea mays*	162459661	35.5/6.82	34/6.70	118	7	MS/MS
13	sequence 5 from patent US 5668007		2492077	19/6.68	19/6.77	189	3	MS
47	type I small heat shock protein 17.7 kDa I2Iisoform	*Solanum lycopersicum*	24954801	18/5.52	17/5.58	203	1	MS
110	cytosolic heat shock protein 90	*Hordeum vulgare*	32765549	80/5.10	80/4.96	219	29	MS
**ATP Interconversion**								
11	atp1	*Triticum aestivum*	81176509	55.6/5.7	54/5.78	195	15	MS/MS
74	Nucleoside diphosphate kinase	*Lolium perenne*	9652119	15/6.36	16/6.3	192	3	MS
**Chaperones**								
1	HSP70	*Hordeum vulgare subsp. vulgare*	476003	*67.1/5.76*	65/5.36	1080	34	MS/MS
31	HSP70	*Hordeum vulgare subsp. vulgare*	476003	67/5.76	67/5.24	170	12	MS
10	cyclophilin-like protein	*Triticum aestivum*	37788308	26.1/9.4	19/6.97	260	10	MS/MS
**Signal Transduction**								
8	G beta-like protein	*Glycine max*	1256608	35/7.62	37/5.97	75	1	MS/MS
26	WD-40 repeat protein	*Arabidopsis thaliana*	21594513	34/4.68	35/7.62	75	1	MS

Table S1. *Cont.*

**Transporting-associated proteins**								
2	ATP-dependent RNA helicase eIF4A	*Zea mays*	1170509	47.2/5.31	52/5.49	815	25	MS/MS
**unknown**								
36	predicted protein	*Physcomitrella patens subsp. patens*	168046982	41.9/5.13	48/5.42	191	11	MS/MS
91	sequence 844 from patent US 6753314	*Oryza sativa*	53971832	16/6.01	65/8.16	161	17	MS

[a] Theor.Mr/pI: molecular mass of predicted protein/p*I* of predicted protein; [b] Exp.Mr/pI: molecular mass of protein on the gel/ p*I* of protein on the gel; [c] Score: statistical probability of true positive identification of the predicted protein calculated by MASCOT with 0.3 peptide tolerance and one allowed missed cleavage (score ≥42 against NCBInr, ≥66 against MSDB, or ≥55 against Swiss-Prot); [d] PN: Number of peptides searched by MS and sequenced by MS/MS; [e] Method: MS = MALDI-TOF, MS/MS = MALDI-TOF/TOF.

Table S2. Differentially expressed non-prolamins under control and drought stress conditions in Janz and Kauz by nonlinear 2-DE (pH 3–10, 18 cm) and MALDI mass spectrometry.

Sport ID	Protein name	Plant species	Accession No (gi:)	Theor. Mr/pI [a]	Score [b]	PN [c]	Method [d]
**carbon metabolism**							
208	ADP-glucose pyrophosphorylase large subunit	*Triticum aestivum*	32812836	58.4/5.89	477	22	MS/MS
209	ADP-glucose pyrophosphorylase large subunit	*Triticum aestivum*	32812836	58.4/5.89	972	28	MS/MS
214	beta amylase	*Triticum aestivum*	32400764	31.1/8.6	567	13	MS/MS
303	aestivum stearoyl-ACP desaturase	*Triticum aestivum*	319739540	44.7/8.15	453	16	MS/MS
304	beta amylase	*Triticum aestivum*	32400764	31.1/8.6	434	13	MS/MS
310	UDP-glucose pyrophosphorylase	*Hordeum vulgare*	6136111	51.8/5.2	1,110	27	MS/MS
311	sucrose synthase type 2	*Triticum aestivum*	3393044	93.1/6.17	809	31	MS/MS
312	sucrose synthase type 2	*Triticum aestivum*	3393044	93.1/6.17	636	31	MS/MS
316	alpha-galactosidase	*Hordeum vulgare subsp. vulgare*	2204226	22.2/6.4	125	7	MS/MS
320	triosephosphat-isomerase	*Triticum aestivum*	11124572	27/5.38	642	14	MS/MS

Table S2. *Cont.*

325	phosphoglucomutase	*Triticum aestivum*	18076790	63/5.66	650	26	MS/MS
327	beta amylase	*Triticum aestivum*	32400764	31.1/8.6	474	15	MS/MS
331	tissue-ubiquitous beta-amylase 2	*Sorghum bicolor*	61006859	57.3/5.46	155	6	MS/MS
**Detoxification and defense**							
319	ascorbate peroxidase	*Hordeum vulgare subsp. vulgare*	15808779	28/5.1	520	11	MS/MS
322	serpin 1	*Triticum aestivum*	224589266	43.3/5.44	985	18	MS/MS
323	Serpin-Z1C	*Triticum aestivum*	75313848	43/5.62	325	11	MS/MS
314	Alpha-amylase/trypsin inhibitor CM3;	*Triticum aestivum*	123957	18.9/5.62	614	8	MS/MS
334	dehydroascorbate reductase	*Triticum aestivum*	28192421	23.5/5.88	812	15	MS/MS
**Photosynthesis**							
201	ribulose-1,5-bisphosphate carboxylase/oxygenase large subunit	*Hordeum erectifolium*	31087877	53.3/6.13	557	25	MS/MS
202	ribulose-1,5-bisphosphate carboxylase/oxygenase large subunit	*Hordeum patagonicum subsp. santacrucense*	31087891	53.4/5.95	839	31	MS/MS
210	Os01g0328700	*Oryza sativa Japonica Group*	115436320	53.0/7.21	257	*13*	MS/MS
313	ribulose-1,5-bisphosphate carboxylase/oxygenase large subunit	*Triticum aestivum*	14017580	53.4/6.22	311	18	MS/MS
**Nitrogen Metabolism**							
203	Alanine aminotransferase 2	*Hordeum vulgare*	1703227	53.4/5.93	567	21	MS/MS
213	Alanine aminotransferase 2	*Hordeum vulgare*	1703227	53.4/5.93	608	23	MS/MS
324	methionine synthase 1 enzyme	*Hordeum vulgare*	68655495	84.9/5.74	483	17	MS/MS
**ATP Interconversion**							
301	F0-F1 ATPase alpha subunit	*Sorghum bicolor*	9408184	47.8/5.96	1,080	22	MS/MS
329	ATP synthase CF1 alpha subunit	*Triticum aestivum*	14017569	55.3/6.11	1,290	32	MS/MS
**Signal Transduction**							
204	Os04g0118400	*Oryza sativa Japonica Group*	115456914	94.9/5.85	252	19	MS/MS

Table S2. *Cont.*

**Unknown**							
205	Os06g0247500	*Oryza sativa Japonica Group*	115467370	61.9/6.01	78	6	MS/MS
317	predicted protein	*Hordeum vulgare subsp. vulgare*	326496084	36.7/6.06	538	14	MS/MS
318	predicted protein	*Hordeum vulgare subsp. vulgare*	326497973	39.1/6.27	428	13	MS/MS
315	predicted protein	*Hordeum vulgare subsp. vulgare*	326510251	86.1/5.81	532	26	MS/MS
328	predicted protein	*Hordeum vulgare subsp. vulgare*	326503994	68.8/6.08	442	16	MS/MS
332	predicted protein	*Hordeum vulgare subsp. vulgare*	326493416	32.8/5.34	194	14	MS/MS
330	hypothetical protein SORBIDRAFT_02g029260	*Sorghum bicolor*	242049776	24.7/6.43	290	12	MS/MS

[a] Theor.Mr/pI: molecular mass of predicted protein/ p*I* of predicted protein; [b] Score: statistical probability of true positive identification of the predicted protein calculated by MASCOT with 0.3 peptide tolerance and one allowed missed cleavage; (score ≥42 against NCBInr, ≥66 against MSDB, or ≥55 against Swiss-Prot). [c] PN: Number of peptides sequenced. [d] Method: MS = MALDI-TOF, MS/MS = MALDI-TOF/TOF.

Table S3. Peptide sequences of identified proteins by MALDI-TOF-TOF MS.

Spot ID	Protein name	Calc. mass[a)]	Observ. mass[b)]	± da	± ppm	Start seq. [c]	End seq. [d]	Sequences [e]	Ion score [f]	C.I. %
1	HSP70	998.5013	998.5214	0.0201	20	86	94	NQAAVNPER	60	99.94
		1052.5596	1052.5812	0.0216	21	302	310	ALSNQHQVR	40	94.275
		1155.6481	1155.6659	0.0178	15	190	201	DAGVIAGLNVAR	92	100
		1464.7118	1464.7228	0.011	8	66	78	ITPSWVGFTDGER	104	100
		1509.8384	1509.8431	0.0047	3	358	371	TQIHEIVLVGGSTR	111	100
		1536.7513	1536.7633	0.012	8	52	65	NGHVEIIANDQGNR	102	100
		1815.9963	1815.9972	0.0009	0	202	218	IINEPTAAAIAYGLDKR	138	100
		1892.9502	1892.9502	0	0	169	185	INDAVVTVPAYFNDAQR	128	100
2	ATPdependent RNA helicase eIF4A	976.5574	976.5557	−0.0017	−2	378	386	GVAINFVTR	46	97.943
		1070.5953	1070.5948	−0.0005	0	177	185	QSLRPDNIK	41	93.189
		1104.6525	1104.6489	−0.0036	−3	377	386	KGVAINFVTR	59	99.896

Table S3. *Cont.*

		1114.6831	1114.6849	0.0018	2	333	342	VLITTDLLAR	75	99.998
		1142.5736	1142.5763	0.0027	2	140	150	VHACVGGTSVR	89	100
		1173.6475	1173.6442	−0.0033	−3	246	255	RDELTLEGIK	54	99.707
		1461.8538	1461.8485	−0.0053	−4	155	169	ILASGVHVVVGTPGR	75	99.998
		1827.9388	1827.9368	−0.002	−1	55	70	GIYAYGFEKPSAIQQR	120	100
7	cytosolic glyceraldehyde-3-phosphate dehydrogenase GAPDH	1184.6423	1184.6376	−0.0047	−4	199	209	AGIALNDHFVK	59	99.9
		1247.6995	1247.6683	−0.0312	−25	149	159	LAKPATYDQIK	41	93.347
		1498.8477	1498.8447	−0.003	−2	135	148	VPTVDVSVVDLTVR	121	100
		2213.0356	2213.0701	0.0345	16	172	191	GILGYVDEDLVSTDFQGD	64	99.967
8	Os01g0686800	1034.5088	1034.5034	−0.0054	−5	193	200	VWNLTNCK	41	95.184
		1248.6583	1248.6569	−0.0014	−1	101	111	LWDLSTGVTTR	79	100
10	cyclophilin-like protein	844.4312	844.4391	0.0079	9	196	202	TPWLDGR	28	11.499
		1590.7719	1590.7672	−0.0047	−3	218	231	TIESSETDRGDRPK	33	67.495
		2035.0496	2035.0465	−0.0031	−2	97	114	IVIGLYGDDVPQTVENFR	114	100
11	atp1	1203.658	1203.6624	0.0044	4	7	17	AAELTTLLESR	44	97.303
14	Serpin-Z2B	925.5214	925.5243	0.0029	3	11	18	LSIAHQTR	56	99.83
		1137.6667	1137.6603	−0.0064	−6	172	181	LVLGNALYFK	71	99.995
		1192.5382	1192.5344	−0.0038	−3	182	191	GAWTDQFDPR	69	99.992
		1372.7068	1372.7004	−0.0064	−5	159	171	DILPAGSIDNTTR	86	100
		1514.7485	1514.7402	−0.0083	−5	125	137	YKAEAQSVDFQTK	100	100
		1665.8595	1665.851	−0.0085	−5	261	274	LSAEPEFLEQHIPR	81	100
		1922.9706	1922.975	0.0044	2	335	353	AFVEVNETGTEAAATTIA	143	100
		2085.155	2085.146	−0.009	−4	152	171	VTTGLIKDILPAGSIDNTT	138	100
15	Serpin-Z2B	925.5214	925.5187	−0.0027	−3	11	18	LSIAHQTR	48	98.903
		1137.6667	1137.6552	−0.0115	−10	172	181	LVLGNALYFK	38	88.377
		1192.5382	1192.5319	−0.0063	−5	182	191	GAWTDQFDPR	57	99.864
		1514.7485	1514.7379	−0.0106	−7	125	137	YKAEAQSVDFQTK	77	99.999

Table S3. *Cont.*

		1665.8595	1665.8508	−.0087	−5	261	274	LSAEPEFLEQHIPR	97	100
		1922.9706	1922.974	0.0034	2	335	353	AFVEVNETGTEAAATTIA	112	100
		2085.155	2085.1526	−0.0024	−1	152	171	VTTGLIKDILPAGSIDNTT	85	100
20	peroxidase 1	958.5064	958.5092	0.0028	3	113	120	AVNDIRDR	32	47.438
		998.5992	998.5987	−0.0005	−1	62	71	DIGLAAGLLR	77	99.998
		1004.4836	1004.4828	−0.0008	−1	34	41	GLSFDFYR	35	75.696
		1486.8047	1486.7958	−0.0089	−6	127	141	GAVVSCADILALAAR	114	100
		1772.9291	1772.9222	−0.0069	−4	142	158	DSVVVSGGPDYRVPLGR	63	99.962
22	beta amylase	1006.554	1006.5618	0.0078	8	211	219	NARPHGINK	48	99.062
		1326.6688	1326.6703	0.0015	1	200	210	YDPTAYNTILR	91	100
23	Os05g0573700	1646.781	1646.7743	−0.0067	−4	61	74	FFVDNGTYLTEQGR	88	100
		1372.7219	1372.7213	−0.0006	0	80	91	VSLAGHEEYIVR	89	100
		1528.6884	1528.692	0.0036	2	491	504	GVAFMVDNCSTTAR	108	100
		1544.6832	1544.6931	0.0099	6	491	504	GVAFMVDNCSTTAR	35	79.084
24	embryo globulin	991.5472	991.5861	0.0389	39	181	188	RPYVFGPR	63	99.966
		1028.5558	1028.5962	0.0404	39	222	230	VAIMEVNPR	57	99.866
27	cytoplasmic aldolase	829.3839	829.3916	0.0077	9	133	139	YYEAGAR	45	97.899
		1346.7314	1346.7264	−0.005	−4	238	249	VAPEVIAEYTVR	87	100
		1474.8264	1474.8239	−0.0025	−2	237	249	KVAPEVIAEYTVR	77	99.999
		1488.8016	1488.7985	−0.0031	−2	25	39	GILAADESTGTIGKR	77	99.999
		1662.8195	1662.8169	−0.0026	−2	40	53	FASINVENVEDNRR	40	94.106
28	23kDa polypeptide of photosystem II	945.5152	945.5126	−0.0026	−3	109	116	EFPGQVLR	50	99.405
29	Os07g0683900	1047.5331	1047.5349	0.0018	2	260	269	HSTGQGHPVK	40	92.813
		1064.4215	1064.4219	0.0004	0	330	337	WCEGDNQR	37	86.586
		1089.5801	1089.5796	−0.0005	0	87	96	HSLGQSHPVK	37	85.626
30	putative r40c2 protein	1064.4215	1064.4224	0.0009	1	272	279	WCEGDNQR	37	87.271
		1089.5801	1089.5813	0.0012	1	29	38	HSLGQSHPVK	53	99.649
		1721.8857	1721.9185	0.0328	19	180	194	IRDEEGYPAFALVNK	86	100

Table S3. *Cont.*

32	embryo globulin	991.5472	991.5847	0.0375	38	181	188	RPYVFGPR	66	99.981
		1028.5558	1028.592	0.0362	35	222	230	VAIMEVNPR	44	97.146
		1269.6117	1269.6616	0.0499	39	37	47	GGHSLQQCVQR	63	99.962
33	beta amylase	1006.554	1006.5599	0.0059	6	211	219	NARPHGINK	53	99.656
		1326.6688	1326.6676	−0.0012	−1	200	210	YDPTAYNTILR	75	99.998
		1474.6777	1474.674	−0.0037	−3	187	199	EGLNMACENALPR	88	100
		1646.781	1646.7755	−0.0055	−3	61	74	FFVDNGTYLTEQGR	98	100
35	polyphenol oxidase	966.4526	966.4545	0.0019	2	80	87	SSPLYDER	45	97.827
		1207.6833	1207.6831	−0.0002	0	133	142	TLLFLGQPYR	31	44.406
		1285.6682	1285.6654	−0.0028	−2	237	247	VRDCLDPAALR	40	93.675
		2156.1194	2156.1245	0.0051	2	265	287	ASGGTPAPATTGTLPATL	162	100
36	predicted protein	976.4483	976.4512	0.0029	3	22	31	AGFAGDDAPR	62	99.953
		1132.527	1132.5308	0.0038	3	200	209	GYSFTTTAER	45	97.571
38	small subunit ADP glucose pyrophosphorylase	900.5261	900.5289	0.0028	3	422	429	AIIDKNAR	29	21.449
		1017.5952	1017.5916	−0.0036	−4	366	374	IHHSVVGLR	69	99.993
		1032.5472	1032.549	0.0018	2	330	338	SAPIYTQPR	62	99.965
		1103.5845	1103.5834	−0.0011	−1	9	18	TFPSPSPSKR	39	92.753
		1256.7109	1256.7067	−0.0042	−3	64	75	AKPAVPLGANYR	49	99.17
		1621.7925	1621.7861	−0.0064	−4	228	242	AMMVDTTILGLDDAR	64	99.978
39	globulin 3B	2089.9058	2089.894	−0.0118	−6	461	478	DQQDEGFVAGPEQQEQ	105	100
40	peroxidase 1	998.5992	998.5756	−0.0236	−24	62	71	DIGLAAGLLR	61	99.939
		1486.8047	1486.7577	−0.047	−32	127	141	GAVVSCADILALAAR	81	100
45	aldose reductase-related protein	1061.5408	1061.5392	−0.0016	−2	72	81	AAMEAGIDRK	56	99.823
		1121.595	1121.5883	−0.0067	−6	223	232	NLAHDPVVEK	43	96.803
		1934.9203	1934.9127	−0.0076	−4	34	52	AGSDTAHSVQTAITEAGY	138	100
46	BAC19.13	1245.6475	1245.6433	−0.0042	−3	151	161	VEEGDVFVVPR	65	99.975

Table S3. *Cont.*

49	globulin 3	1822.8752	1822.8661	−0.0091	−5	489	504	GSSNLQVVCFEINAER	127	100
		1906.0182	1906.0131	−0.0051	−3	470	488	GSAFVVPPGHPVVEIASS	113	100
		2222.0618	2222.0579	−0.0039	−2	489	507	GSSNLQVVCFEINAERNE	47	99.96
50	globulin 3	1822.8752	1822.8713	−0.0039	−2	489	504	GSSNLQVVCFEINAER	118	100
		1906.0182	1906.0134	−0.0048	−3	470	488	GSAFVVPPGHPVVEIASS	94	100
		2222.0618	2222.0667	0.0049	2	489	507	GSSNLQVVCFEINAERNE	71	100
53	glutamine synthetase isoform GSr1	960.4785	960.4813	0.0028	3	170	177	DIVDAHYK	54	99.752
		1440.7693	1440.7731	0.0038	3	39	52	TVNGPITDASQLPK	97	100
		1552.7251	1552.7286	0.0035	2	277	290	HAQHIAAYGEGNER	121	100
		1583.7812	1583.7775	−0.0037	−2	292	305	LTGHHETADINTFK	89	100
55	Os05g0453700	1400.6838	1400.682	−0.0018	−1	13	25	IGVAMDYSASSKR	48	98.742
56	Catalase isozyme 1	974.5054	974.508	0.0026	3	103	110	FSTVVHER	68	99.988
		1034.5378	1034.5367	−0.0011	−1	51	58	LAQFDRER	31	33.49
		1127.5804	1127.5795	−0.0009	−1	111	120	GSPETLRDPR	44	97.291
		1136.6787	1136.6774	−0.0013	−1	92	102	APGVQTPVIVR	54	99.667
58	annexin2	1773.8767	1773.8855	0.0088	5	132	147	SLEEDVAAHVTGDFRK	62	99.954
59	putative proteasome subunit alpha type 3	826.4457	826.449	0.0033	4	160	167	YFGAALGK	42	95.126
		1019.523	1019.5229	−0.0001	0	102	110	VYGEPMPVK	30	15.486
		1040.5411	1040.5355	−0.0056	−5	21	29	VFQVEYAGK	38	86.971
		1217.6559	1217.6532	−0.0027	−2	42	52	CKDGIVLGVEK	57	99.857
61	triticin precursor	885.5152	885.5184	0.0032	4	50	57	LQASTPLR	31	23.932
		931.4632	931.4641	0.0009	1	147	153	FKDEHQK	30	6.416
63	glucose and ribitol dehydrogenase homolog - barley	1201.6536	1201.6584	0.0048	4	42	54	VALVTGGDSGIGR	56	99.848
		1711.8246	1711.8544	0.0298	17	74	88	GHEDKDAEETLQALR	78	100

Table S3. *Cont.*

64	avenin-like protein	818.4553	818.4599	0.0046	6	248	254	MSLQALR	29	93.405
		1245.6587	1245.661	0.0023	2	202	211	QLSQIPEQFR	77	100
		1381.7006	1381.6985	−0.0021	−2	212	223	CQAIHNVAEAIR	90	100
		2925.406	2925.4216	0.0156	5	224	247	QQQPQQQWQGMYQPQ	98	100
65	beta amylase	1006.554	1006.5628	0.0088	9	211	219	NARPHGINK	33	68.243
		1299.582	1299.5846	0.0026	2	151	161	ASLNFTCAEMR	79	100
		1326.6688	1326.6726	0.0038	3	200	210	YDPTAYNTILR	82	100
		1474.6777	1474.6779	0.0002	0	187	199	EGLNMACENALPR	111	100
		1646.781	1646.7781	−0.0029	−2	61	74	FFVDNGTYLTEQGR	92	100
		1668.7952	1668.7903	−0.0049	−3	33	47	AAAAMVGHPEWEFPR	39	91.393
66	Alpha-amylase/trypsin inhibitor CM3	1010.52	1010.5317	0.0117	12	37	44	TNLLPHCR	47	98.828
		1110.5038	1110.5115	0.0077	7	133	140	EMQWDFVR	67	99.987
		1126.4987	1126.5039	0.0052	5	133	140	EMQWDFVR	49	99.247
		1698.9214	1698.9071	−0.0143	−8	101	115	YFIALPVPSQPVDPR	64	99.977
		1727.8381	1727.8246	−0.0135	−8	116	132	SGNVGESGLIDLPGCPR	155	100
		1957.8564	1957.8285	−0.0279	−14	81	95	LYCCQELAEISQQCR	119	100
67	Os01g0743500	1315.7217	1315.7161	−0.0056	−4	175	186	SIQVIVVTDGER	40	93.573
		1539.6606	1539.6599	−0.0007	0	571	582	YAESCMYSPIYR	63	99.965
69	Formate dehydrogenase	817.4526	817.4579	0.0053	6	192	200	TVGTVGAGR	44	97.914
		955.5683	955.5684	0.0001	0	275	283	GVIIVNNAR	54	99.803
		1209.6124	1209.6104	−0.002	−2	174	184	GEWNVAGIAHR	52	99.694
		1292.6998	1292.6926	−0.0072	−6	163	173	NFLPGYQQVVK	48	99.228
70	xylose isomerase	1358.7162	1358.7085	−0.0077	−6	416	427	LIEDGSLDELVR	78	99.999
		1568.7737	1568.7596	−0.0141	−9	235	247	EGYQTLLNTDMKR	62	99.948
		2672.2053	2672.1926	−0.0127	−5	308	330	INVECNHATLSGHSCHHE	99	100

Table S3. *Cont.*

71	NADPH producing dehydrogenase of the oxidative pentose phosphate pathway	1052.5524	1052.5527	0.0003	0	26	34	GFPISVYNR	39	91.251
		1064.5735	1064.5732	−0.0003	0	292	300	FLSGLKDER	49	99.147
		1109.6426	1109.6406	−0.002	−2	441	450	LPANLVQAQR	38	90.07
		1258.5488	1258.5476	−0.0012	−1	451	460	DYFGAHTYER	62	99.957
		1380.7706	1380.7668	−0.0038	−3	439	450	DRLPANLVQAQR	48	98.836
		1665.8265	1665.8234	−0.0031	−2	122	138	GLLYLGMGVSGGEEGAR	63	99.963
		1681.8214	1681.8153	−0.0061	−4	122	138	GLLYLGMGVSGGEEGAR	44	97.5
72	Serpin-Z2B	925.5214	925.4877	−0.0337	−36	11	18	LSIAHQTR	32	80.575
		1514.7485	1514.6855	−0.063	−42	125	137	YKAEAQSVDFQTK	90	100
78	embryo globulin	1028.5558	1028.5631	0.0073	7	222	230	VAIMEVNPR	57	99.838
		1360.7219	1360.72	−0.0019	−1	387	397	DTFNLLEQRPK	60	99.923
79	embryo globulin	832.4159	832.3878	−0.0281	−34	355	361	ASEEQLR	31	34.951
		991.5472	991.509	−0.0382	−39	181	188	RPYVFGPR	30	27.349
		1028.5558	1028.515	−0.0408	−40	222	230	VAIMEVNPR	52	99.55
		1360.7219	1360.6699	−0.052	−38	387	397	DTFNLLEQRPK	69	99.991
80	embryo globulin	837.4101	837.3801	−0.03	−36	405	411	LYEADAR	43	96.557
		991.5472	991.5067	−0.0405	−41	181	188	RPYVFGPR	31	39.615
		1028.5558	1028.5132	−0.0426	−41	222	230	VAIMEVNPR	55	99.784
81	embryo globulin	832.4159	832.3873	−0.0286	−34	355	361	ASEEQLR	32	59.48
		1360.7219	1360.6752	−0.0467	−34	387	397	DTFNLLEQRPK	73	99.997
82	embryo globulin	991.5472	991.5135	−0.0337	−34	181	188	RPYVFGPR	38	90.769
		1028.5558	1028.519	−0.0368	−36	222	230	VAIMEVNPR	50	99.36
		1360.7219	1360.6748	−0.0471	−35	387	397	DTFNLLEQRPK	76	99.998
86	embryo globulin	837.4101	837.4385	0.0284	34	405	411	LYEADAR	46	97.995
		991.5472	991.5724	0.0252	25	181	188	RPYVFGPR	35	74.52
		1005.4959	1005.5253	0.0294	29	329	336	TSDERLER	30	16.976

Table S3. *Cont.*

		1028.5558	1028.585	0.0292	28	222	230	VAIMEVNPR	54	99.717
89	globulin 3	1685.8606	1685.8547	−0.0059	−3	520	534	LDDPAQELTFGRPAR	76	100
		1906.0182	1906.0074	−0.0108	−6	470	488	GSAFVVPPGHPVVEIASS	94	100
		2426.0967	2426.0835	−0.0132	−5	542	562	AKDQQDEGFVAGPEQQ	140	100
90	globulin 3	1906.0182	1905.9873	−0.0309	−16	470	488	GSAFVVPPGHPVVEIASS	114	100
		2222.0618	2222.0339	−0.0279	−13	489	507	GSSNLQVVCFEINAERNE	64	99.999
97	peroxidase 1	958.5064	958.5115	0.0051	5	113	120	AVNDIRDR	35	76.675
		998.5992	998.6032	0.004	4	62	71	DIGLAAGLLR	66	99.981
		1004.4836	1004.4861	0.0025	2	34	41	GLSFDFYR	36	80.732
		1772.9291	1772.9231	−0.006	−3	142	158	DSVVVSGGPDYRVPLGR	67	99.985
99	Chain A, Crystal Structure Of Xylanase Inhibitor Protein (Xip-I) From Wheat	977.5414	977.5448	0.0034	3	175	183	ALATGIFER	42	95.801
		1150.6005	1150.604	0.0035	3	5	14	TGQVTVFWGR	37	87.054
		1184.563	1184.5685	0.0055	5	164	174	CGYPPAAHVGR	70	99.994
		1308.6947	1308.6896	−0.0051	−4	235	246	NVYYGVAPVAQK	40	93.527
		1403.8118	1403.8142	0.0024	2	150	163	GGPGKPLHLTATVR	105	100
100	Basic endochitinase C	1743.8562	1743.8577	0.0015	1	138	153	GPIQLSHNYNYGPAGR	93	100
		2610.1389	2610.1709	0.032	12	245	266	YCDILGVGYGDNLDCYN	150	100
101	triticin precursor	826.3512	826.3547	0.0035	4	44	49	ECTFNR	29	20.532
		1381.7012	1381.7037	0.0025	2	200	210	HKEFLFAGNYR	57	99.884
		1639.7671	1639.7689	0.0018	1	132	146	YGQSQSVQGQSQSQK	103	100
		1908.9199	1908.9244	0.0045	2	211	227	SSQLHSSQNIFSGFDVR	144	100
		2062.8989	2062.9048	0.0059	3	61	77	SQAGLTEYFDEENEQFR	160	100
102	Os01g0760600	848.4625	848.4718	0.0093	11	168	175	VGGEFLAR	51	99.49
		1448.7527	1448.7416	−0.0111	−8	154	167	VATVQCLSGTGSLR	79	100
		1530.7911	1530.7767	−0.0144	−9	140	153	LIFGADSPAIQENR	98	100
114	peroxidase 1	998.5992	998.5979	−0.0013	−1	62	71	DIGLAAGLLR	58	99.906
		1772.9291	1772.9325	0.0034	2	142	158	DSVVVSGGPDYRVPLGR	51	99.514

Table S3. *Cont.*

130	triticin precursor	826.3512	826.3613	0.0101	12	44	49	ECTFNR	33	70.205
		885.5152	885.5208	0.0056	6	50	57	LQASTPLR	39	92.653
		931.4632	931.4673	0.0041	4	147	153	FKDEHQK	30	39.307
		1263.7208	1263.7168	−0.004	−3	259	268	FLKPVFTQQR	59	99.918
		1381.7012	1381.6947	−0.0065	−5	200	210	HKEFLFAGNYR	91	100
		1639.7671	1639.7668	−0.0003	0	132	146	YGQSQSVQGQSQSQK	133	100
		1908.9199	1908.9104	−0.0095	−5	211	227	SSQLHSSQNIFSGFDVR	143	100
		2062.8989	2062.8926	−0.0063	−3	61	77	SQAGLTEYFDEENEQFR	141	100
131	triticin precursor	826.3512	826.3593	0.0081	10	44	49	ECTFNR	31	51.425
		885.5152	885.5201	0.0049	6	50	57	LQASTPLR	40	93.842
		923.4767	923.4813	0.0046	5	78	85	CTGVFAIR	32	64.073
		1079.5779	1079.5862	0.0083	8	78	86	CTGVFAIRR	38	91.14
		1263.7208	1263.7285	0.0077	6	259	268	FLKPVFTQQR	53	99.668
		1381.7012	1381.7091	0.0079	6	200	210	HKEFLFAGNYR	91	100
		1639.7671	1639.7738	0.0067	4	132	146	YGQSQSVQGQSQSQK	133	100
		1908.9199	1908.9321	0.0122	6	211	227	SSQLHSSQNIFSGFDVR	135	100
		2062.8989	2062.9143	0.0154	7	61	77	SQAGLTEYFDEENEQFR	166	100
132	triticin precursor	826.3512	826.3615	0.0103	12	44	49	ECTFNR	33	67.829
		885.5152	885.5229	0.0077	9	50	57	LQASTPLR	52	99.618
		931.4632	931.4673	0.0041	4	147	153	FKDEHQK	37	85.463
		1079.5779	1079.5791	0.0012	1	78	86	CTGVFAIRR	30	39.957
		1263.7208	1263.7192	−0.0016	−1	259	268	FLKPVFTQQR	39	92.493
		1381.7012	1381.6997	−0.0015	−1	200	210	HKEFLFAGNYR	91	100
		1639.7671	1639.7721	0.005	3	132	146	YGQSQSVQGQSQSQK	133	100
		1908.9199	1908.9265	0.0066	3	211	227	SSQLHSSQNIFSGFDVR	146	100
		2062.8989	2062.9092	0.0103	5	61	77	SQAGLTEYFDEENEQFR	137	100
133	triticin precursor	885.5152	885.5148	−0.0004	0	50	57	LQASTPLR	47	98.462
		931.4632	931.4602	−0.003	−3	147	153	FKDEHQK	33	59.927

Table S3. *Cont.*

		1381.7012	1381.6956	−0.0056	−4	200	210	HKEFLFAGNYR	75	99.998
		1908.9199	1908.9254	0.0055	3	211	227	SSQLHSSQNIFSGFDVR	144	100
		2062.8989	2062.9116	0.0127	6	61	77	SQAGLTEYFDEENEQFR	143	100
135	Os01g0760600	848.4625	848.4687	0.0062	7	168	175	VGGEFLAR	29	14.859
		1448.7527	1448.7505	−0.0022	−2	154	167	VATVQCLSGTGSLR	51	99.396
		1530.7911	1530.7849	−0.0062	−4	140	153	LIFGADSPAIQENR	40	93.376
136	0.19 dimeric alpha-amylase inhibitor	1162.6249	1162.6119	−0.013	−11	90	100	LTAASITAVCR	53	99.815
		1570.8007	1570.7505	−0.0502	−32	26	39	LQCNGSQVPEAVLR	108	100
		1617.8993	1617.8418	−0.0575	−36	86	100	EVVKLTAASITAVCR	94	100
		1663.8361	1663.7784	−0.0577	−35	101	116	LPIVVDASGDGAYVCK	137	100
		1840.7412	1840.661	−0.0802	−44	40	53	DCCQQLADISEWCR	129	100
138	Endogenous alpha-amylase/subtilisin inhibitor	1006.5316	1006.5275	−0.0041	−4	122	129	ENAFRIEK	59	99.919
		1182.5426	1182.5479	0.0053	4	130	139	YSGAEVHEYK	51	99.455
		1318.6433	1318.6582	0.0149	11	27	40	AHGGGLTMAPGHGR	82	100
		1366.675	1366.6927	0.0177	13	15	26	ADANYYVLPANR	74	99.997
		1431.8066	1431.828	0.0214	15	61	74	IAPHGGAPSDKIIR	99	100
		1506.7006	1506.7258	0.0252	17	42	54	CPLFVSQEADGQR	107	100
		1574.8398	1574.873	0.0332	21	107	121	HVITGPVRDPSPSGR	60	99.929
		2144.0554	2144.1357	0.0803	37	42	60	CPLFVSQEADGQRDGLP	51	99.401
201	ribulose-1,5-bisphosphate carboxylase/oxygenase large subunit	1187.6644	1187.6587	−0.0057	−5	286	295	DNGLLLHIHR	65	99.958
		1339.7006	1339.6816	−0.019	−14	9	21	AGVGFQAGVKDYK	71	99.988
		1465.7546	1465.7396	−0.015	−10	147	159	TFQGPPHGIQVER	103	100
202	ribulose-1,5-bisphosphate carboxylase/oxygenase large subunit	898.41	898.4231	0.0131	15	306	312	NHGMHFR	44	92.822
		971.4073	971.4184	0.0111	11	188	194	ACYECLR	46	95.225
		985.5789	985.5862	0.0073	7	132	139	ALRLEDLR	45	94.39

Table S3. *Cont.*

		1021.5312	1021.5359	0.0047	5	33	41	DTDILAAFR	61	99.87
		1116.583	1116.584	0.001	1	422	431	VALEACVQAR	53	99.145
		1187.6644	1187.6661	0.0017	1	286	295	DNGLLLHIHR	91	100
		1465.7546	1465.7429	−0.0117	−8	147	159	TFQGPPHGIQVER	116	100
		1502.8512	1502.8303	−0.0209	−14	165	177	YGRPLLGCTIKPK	43	91.509
203	Alanine aminotransferase 2	1022.5741	1022.5818	0.0077	8	23	31	GEIVIHAQR	34	70.108
		1089.4783	1089.4875	0.0092	8	300	308	GYYGECGKR	51	99.392
		1096.5634	1096.5701	0.0067	6	87	96	TLFSADSISR	37	85.889
		1219.6066	1219.6086	0.002	2	108	119	ATGAYSHSQGIK	86	100
		1243.7004	1243.7008	0.0004	0	362	373	AEKDGILASLAR	81	100
		1407.7379	1407.734	−0.0039	−3	415	427	AANKAPDAFYALR	104	100
204	Os04g0118400	890.5095	890.5095	0	0	483	490	FSVSPVVR	36	78.877
		1346.7791	1346.7673	−0.0118	−9	713	724	VIYASQLTAKPR	34	70.368
205	Os06g0247500	1339.6853	1339.682	−0.0033	−2	26	37	LASVYSEVQTSR	35	82.551
208	ADP-glucose pyrophosphorylase large subunit	952.4887	952.4886	−0.0001	0	380	387	TPFFTSPR	42	95.373
		1023.5945	1023.5914	−0.0031	−3	415	423	IEHSIIGVR	38	87.403
		1261.6359	1261.6361	0.0002	0	111	122	ATPAVPIGGCYR	65	99.975
		1306.6243	1306.6259	0.0016	1	468	478	ISNCIIDMNAR	31	46.138
		1515.7438	1515.7467	0.0029	2	251	264	ASEYGLVKFDSSGR	61	99.938
		2055.9399	2055.9553	0.0154	7	232	250	HVDDNADITLSCAPVGES	57	99.837
209	ADP-glucose pyrophosphorylase large subunit	952.4887	952.4885	−0.0002	0	380	387	TPFFTSPR	51	99.241
		1023.5945	1023.5903	−0.0042	−4	415	423	IEHSIIGVR	56	99.762
		1261.6359	1261.6304	−0.0055	−4	111	122	ATPAVPIGGCYR	66	99.979
		1306.6243	1306.6161	−0.0082	−6	468	478	ISNCIIDMNAR	80	100
		1440.7628	1440.7571	−0.0057	−4	412	423	ECKIEHSIIGVR	72	99.995
		1515.7438	1515.7361	−0.0077	−5	251	264	ASEYGLVKFDSSGR	106	100

Table S3. *Cont.*

		1646.8174	1646.8071	−0.0103	−6	321	334	YAELHDFGSEILPR	82	100
		2371.1924	2371.197	0.0046	2	54	77	GPAATGAQCVLTSDASP	177	100
210	Os01g0328700	969.4901	969.4843	−0.0058	−6	101	109	SSFAHHGVK	62	99.963
		1523.7423	1523.7343	−0.008	−5	88	100	ALLHSSHMYHEAK	69	99.993
		1539.7373	1539.7253	−0.012	−8	88	100	ALLHSSHMYHEAK	51	99.582
213	Alanine aminotransferase 2	1022.5741	1022.5797	0.0056	5	23	31	GEIVIHAQR	41	94.869
		1089.4783	1089.486	0.0077	7	300	308	GYYGECGKR	50	99.373
		1096.5634	1096.5677	0.0043	4	87	96	TLFSADSISR	37	84.716
		1219.6066	1219.6078	0.0012	1	108	119	ATGAYSHSQGIK	78	99.999
		1243.7004	1243.7026	0.0022	2	362	373	AEKDGILASLAR	89	100
		1407.7379	1407.7416	0.0037	3	415	427	AANKAPDAFYALR	113	100
214	beta amylase	1006.554	1006.5582	0.0042	4	211	219	NARPHGINK	39	91.145
		1299.582	1299.5801	−0.0019	−1	151	161	ASLNFTCAEMR	70	99.993
		1326.6688	1326.6675	−0.0013	−1	200	210	YDPTAYNTILR	79	100
		1474.6777	1474.676	−0.0017	−1	187	199	EGLNMACENALPR	105	100
		1646.781	1646.7767	−0.0043	−3	61	74	FFVDNGTYLTEQGR	85	100
		1668.7952	1668.7894	−0.0058	−3	33	47	AAAAMVGHPEWEFPR	52	99.52
301	F0-F1 ATPase alpha subunit	1026.5942	1026.6088	0.0146	14	143	152	AVDSLVPIGR	54	99.003
		1438.8489	1438.8104	−0.0385	−27	352	365	GIRPAINVGLSVSR	43	87.956
		1537.7434	1537.7026	−0.0408	−27	284	296	EAFPGDVFYLHSR	119	100
		1664.8789	1664.8187	−0.0602	−36	377	390	QVCGSLKLELAQYR	91	100
		1834.8429	1834.7703	−0.0726	−40	7	21	MTNFYTNFQVDEIGR	125	100
		1850.8378	1850.7521	−0.0857	−46	7	21	MTNFYTNFQVDEIGR	153	100
		2025.1267	2025.0051	−0.1216	−60	416	432	LTEVLKQPQYEPLPIEK	80	99.997
		2157.0532	2156.916	−0.1372	−64	32	51	VYGLNEIQAGEMVEFAS	107	100
		2308.1567	2308.0295	−0.1272	−55	391	412	EVAAFAQFGSDLDAATQ	221	100
303	aestivum stearoyl-ACP desaturase	1410.7264	1410.7544	0.028	20	325	336	DYADILEFLVGR	107	100
		1573.8367	1573.8685	0.0318	20	247	260	LAQICGIIASDEKR	68	99.951

Table S3. *Cont.*

		2078.9819	2079.0569	0.075	36	210	226	TENNPYLGFIYTSFQER	145	100
304	beta amylase	1326.6688	1326.6752	0.0064	5	200	210	YDPTAYNTILR	64	99.873
		1646.781	1646.7843	0.0033	2	61	74	FFVDNGTYLTEQGR	98	100
		1668.7952	1668.8022	0.007	4	33	47	AAAAMVGHPEWEFPR	35	13.627
		1684.79	1684.79	0	0	33	47	AAAAMVGHPEWEFPR	49	96.332
		2013.9778	2013.9912	0.0134	7	119	136	VPSHAAEITAGYYNLHDR	113	100
310	UDP-glucose pyrophosphorylase	1052.5371	1052.5259	−0.0112	−11	261	270	GGTLISYEGR	61	99.799
		1312.7583	1312.7523	−0.006	−5	333	345	VLQLETAAGAAIR	105	100
		1358.7566	1358.7385	−0.0181	−13	172	183	IVTEDFLPLPSK	74	99.99
		1769.9796	1769.9667	−0.0129	−7	391	406	VKPSNPSIELGPEFKK	106	100
		1949.0338	1949.0333	−0.0005	0	22	39	LGEISENEKAGFISLVSR	78	99.995
		1967.0121	1967.0092	−0.0029	−1	218	235	EYVFVANSDNLGAIVDIK	126	100
		2198.0261	2198.0547	0.0286	13	154	171	YSNSNIEIHTFNQSQYPR	120	100
		2454.3127	2454.3572	0.0445	18	365	386	ATSDLLLVQSDLYTLVDG	134	100
311	sucrose synthase type 2	1320.7158	1320.7036	−0.0122	−9	67	77	LKDTAFEDLLR	67	99.934
		1425.6904	1425.6851	−0.0053	−4	343	355	LLPDAHGTTCGQR	65	99.889
		1779.9423	1779.9354	−0.0069	−4	602	617	LQELVNLVVVCGDHGK	123	100
		1915.9873	1916.0013	0.014	7	20	37	IGDSLSAHTNELVAVFSR	145	100
		2393.1633	2393.2205	0.0572	24	215	235	KAETHLSGLPADTPYSEF	93	100
312	sucrose synthase type 2	1320.7158	1320.6997	−0.0161	−12	67	77	LKDTAFEDLLR	81	99.997
		1779.9423	1779.9303	−0.012	−7	602	617	LQELVNLVVVCGDHGK	108	100
		1915.9873	1915.9974	0.0101	5	20	37	IGDSLSAHTNELVAVFSR	133	100
		2393.1633	2393.2087	0.0454	19	215	235	KAETHLSGLPADTPYSEF	48	94.276
313	ribulose-1,5-bisphosphate carboxylase/oxygenase large subunit	1465.7546	1465.7535	−0.0011	−1	147	159	TFQGPPHGIQVER	95	100
		2169.9871	2170.0032	0.0161	7	195	213	GGLDFTKDDENVNSQPF	63	99.753
		2185.9819	2185.9734	−0.0085	−4	195	213	GGLDFTKDDENVNSQPF	39	38.504
316	alpha-galactosidase	1113.6085	1113.6025	−0.006	−5	84	93	APLLIGCDVR	74	99.991

Table S3. *Cont.*

317	predicted protein	1262.674	1262.682	0.008	6	101	111	LWDLSTGLTTR	62	99.808
		1321.6747	1321.6875	0.0128	10	119	130	DVISVAFSVDNR	101	100
		1605.896	1605.9014	0.0054	3	282	295	HVVQDLKPEVQVSK	93	100
		2453.2419	2453.3022	0.0603	25	16	39	GHNDVVTAIAAPIDNSPYI	152	100
318	predicted protein	1169.5256	1169.5424	0.0168	14	35	44	ADEGYCLSVR	53	98.65
		1858.9082	1858.9468	0.0386	21	174	191	ILPWGEEAYAGGSANAP	152	100
		2499.2197	2499.3049	0.0852	34	303	324	MVNNIYLNFDALHGDKEH	95	100
		2515.2146	2515.2698	0.0552	22	303	324	MVNNIYLNFDALHGDKEH	107	100
319	ascorbate peroxidase	1249.6172	1249.6163	−0.0009	−1	121	131	QDKPEPPPEGR	55	99.14
		1309.6495	1309.645	−0.0045	−3	132	143	LPDATQGSDHLR	101	100
		2046.908	2046.855	−0.053	−26	225	242	YAADEDAFFADYAEAHLK	131	100
320	Triosephosphat-isomerase	954.4832	954.4866	0.0034	4	5	12	FFVGGNWK	44	88.452
		1289.6332	1289.6414	0.0082	6	195	206	TNVSPEVAESTR	57	99.37
		1374.7046	1374.7117	0.0071	5	124	135	VIACVGETLEQR	98	100
		1415.6359	1415.6383	0.0024	2	101	113	SLMGESSEFVGEK	60	99.68
		1590.8347	1590.8466	0.0119	7	176	190	VASPAQAQEVHANLR	88	100
		1811.9585	1811.9727	0.0142	8	56	70	LRPEIQVAAQNCWVK	83	99.999
		2133.1199	2133.1438	0.0239	11	176	194	VASPAQAQEVHANLRDW	75	99.99
322	serpin 1	925.5214	925.5332	0.0118	13	11	18	LSIAHQTR	49	95.731
		1151.6824	1151.6813	−0.0011	−1	172	181	LVLANALYFK	74	99.988
		1176.5896	1176.5977	0.0081	7	262	271	LSAEPDFLER	94	100
		1292.7097	1292.7112	0.0015	1	290	301	ISFGIEASDLLK	80	99.997
		1544.7592	1544.7596	0.0004	0	125	137	YKAETQSVDFQTK	90	100
		2685.3955	2685.4629	0.0674	25	33	61	SAASNAAFSPVSLHSALS	213	100
		2720.3525	2720.3843	0.0318	12	329	354	VSSVFHQAFVEVNEQGT	218	100
323	Serpin-Z1C	925.5214	925.5155	−0.0059	−6	11	18	LSIAHQTR	38	60.779
		1176.5896	1176.5905	0.0009	1	261	270	LSAEPDFLER	87	100
		1627.8578	1627.8511	−0.0067	−4	287	300	FKISFETEASDLLK	80	99.998

Table S3. *Cont.*

314	Alpha-amylase/trypsin inhibitor CM3;	1110.5038	1110.519	0.0152	14	133	140	EMQWDFVR	56	99.188
		1126.4987	1126.5079	0.0092	8	133	140	EMQWDFVR	50	97.125
		1698.9214	1698.9276	0.0062	4	101	115	YFIALPVPSQPVDPR	83	99.999
		1727.8381	1727.8497	0.0116	7	116	132	SGNVGESGLIDLPGCPR	146	100
		1876.0222	1876.0356	0.0134	7	141	157	LLVAPGQCNLATIHNVR	119	100
		1957.8564	1957.8749	0.0185	9	81	95	LYCCQELAEISQQCR	116	100
315	predicted protein	1786.9375	1786.9623	0.0248	14	691	706	AYLPVIESFGFSSTLR	112	100
		2206.0122	2206.0635	0.0513	23	83	100	CFLELQVEGEEAYQTFS	41	78.529
		2384.188	2384.2451	0.0571	24	269	288	YRVENLYEGPLDDIYANA	61	99.803
		2646.3479	2646.3503	0.0024	1	41	64	ITDGALVVVDCIEGVCVQ	136	100
324	methionine synthase 1 enzyme [Hordeum vulgare	1096.5786	1096.5931	0.0145	13	287	296	YLFAGVVDGR	44	91.127
		2043.0295	2043.0526	0.0231	11	685	704	EGVVYGAGIGPGVYDIHS	154	100
		2268.1719	2268.2031	0.0312	14	590	610	EVEDLEAGGIQVIQIDEAA	180	100
325	phosphoglucomutase	1747.8973	1747.9086	0.0113	6	9	24	ETKPYEGQKPGTSGLR	64	99.916
		1846.8582	1846.8594	0.0012	1	222	238	FSFCFDGLHGVAGAYAK	113	100
		1918.8414	1918.8523	0.0109	6	285	303	TSNVEPPEFGAAADGDA	41	81.954
		2012.1176	2012.137	0.0194	10	86	104	VWVGQDSLLSTPAVSAII	55	99.242
		2168.2188	2168.2332	0.0144	7	85	104	RVWVGQDSLLSTPAVSAI	38	65.929
		2301.1511	2301.1521	0.001	0	114	136	ATGAFILTASHNPGGPTE	90	100
327	beta amylase [Triticum aestivum]	1326.6688	1326.6779	0.0091	7	200	210	YDPTAYNTILR	75	99.99
		1646.781	1646.7937	0.0127	8	61	74	FFVDNGTYLTEQGR	98	100
		1668.7952	1668.8097	0.0145	9	33	47	AAAAMVGHPEWEFPR	64	99.862
		1684.79	1684.7947	0.0047	3	33	47	AAAAMVGHPEWEFPR	43	86.173
		2013.9778	2013.9999	0.0221	11	119	136	VPSHAAEITAGYYNLHDR	101	100
328	predicted protein	1473.8424	1473.882	0.0396	27	429	442	LGANSLLDIVVFGR	110	100
		1518.7336	1518.7769	0.0433	29	585	596	HSLGYWEDEKVR	47	94.326
		1607.8428	1607.89	0.0472	29	124	137	AVIELENYGLPFSR	89	100

Table S3. *Cont.*

329	ATP synthase CF1 alpha subunit	1115.5691	1115.5635	−0.0056	−5	492	500	EAIQEQLER	45	90.272
		1416.7845	1416.7795	−0.005	−4	95	107	IAQIPVSEAYLGR	103	100
		1507.7639	1507.7437	−0.0202	−13	444	456	GYLDSLEIEQVNK	71	99.976
		1519.8051	1519.8019	−0.0032	−2	189	202	GQGVICVYVAIGQR	116	100
		1553.7384	1553.7358	−0.0026	−2	285	297	EAYPGDVFYLHSR	107	100
		1986.0656	1986.0521	−0.0135	−7	464	480	HLKDTKPQFQEIISSSK	110	100
		2121.0896	2121.1042	0.0146	7	42	61	IIGLGEIMSGELVEFAEGT	59	99.65
		2137.0845	2137.0798	−0.0047	−2	42	61	IIGLGEIMSGELVEFAEGT	75	99.991
		2167.208	2167.2136	0.0056	3	108	128	VVNALAKPIDGKGEIIASE	148	100
		2316.1831	2316.2075	0.0244	11	423	443	QSQANPLPVEEQIATIYT	160	100
330	hypothetical protein SORBIDRAFT_02g029260	1381.6781	1381.6759	−0.0022	−2	54	66	CVLASSGFQGDIK	53	98.404
		1465.7659	1465.7765	0.0106	7	76	86	ELLYQHQHNKR	71	99.977
		2023.9801	2023.9877	0.0076	4	105	122	FFPYYAFNVLGGLDSEG	56	99.347
331	tissue-ubiquitous beta-amylase 2[Sorghum bicolor]	2087.0557	2087.093	0.0373	18	129	146	NIEYLTLGVDDQPLFHGR	105	100
332	predicted protein	1278.6365	1278.6577	0.0212	17	267	277	ITSFLDPDGWK	64	99.908
334	dehydroascorbate reductase	1202.682	1202.6826	0.0006	0	102	111	IFSTFVTFLK	56	99.272
		1497.8213	1497.8207	−0.0006	0	157	168	LYHLQVALEHFK	82	99.998
		1574.8578	1574.855	−0.0028	−2	40	52	LIDVSNKPDWFLK	104	100
		1607.8639	1607.8619	−0.002	−1	122	135	ALVDELQALEEHLK	68	99.945
		1827.8444	1827.8525	0.0081	4	8	24	AAVGHPDTLGDCPFSQR	140	100
		2021.0338	2021.0532	0.0194	10	172	189	VPETLTSVHAYTEALFSR	134	100
		2392.2295	2392.271	0.0415	17	169	189	GWKVPETLTSVHAYTEA	70	99.966

[a] Calculated molecular mass of the predicted protein; [b] Observed molecular mass of the predicted protein; [c] Start position of the identified peptide fragment in the protein sequence; [d] End position of the identified peptide fragment in the protein sequence; [e] Multiple matches to peptides with the same primary sequence count; [f] Threshold ion score of credible identification.
